# *Tetrahymena thermophila* Predation Enhances Environmental Adaptation of the Carp Pathogenic Strain *Aeromonas hydrophila* NJ-35

**DOI:** 10.3389/fcimb.2018.00076

**Published:** 2018-03-14

**Authors:** Jin Liu, Yuhao Dong, Nannan Wang, Shougang Li, Yuanyuan Yang, Yao Wang, Furqan Awan, Chengping Lu, Yongjie Liu

**Affiliations:** Joint International Research Laboratory of Animal Health and Food Safety, College of Veterinary Medicine, Nanjing Agricultural University, Nanjing, China

**Keywords:** *Aeromonas hydrophila*, *Tetrahymena*, predation, adaptive traits, environmental adaptation

## Abstract

Persistence of *Aeromonas hydrophila* in aquatic environments is the principle cause of fish hemorrhagic septicemia. Protistan predation has been considered to be a strong driving force for the evolution of bacterial defense strategies. In this study, we investigated the adaptive traits of *A. hydrophila* NJ-35, a carp pathogenic strain, in response to *Tetrahymena thermophila* predation. After subculturing with *Tetrahymena*, over 70% of *A. hydrophila* colonies were small colony variants (SCVs). The SCVs displayed enhanced biofilm formation, adhesion, fitness, and resistance to bacteriophage infection and oxidative stress as compared to the non-*Tetrahymena-*exposed strains. In contrast, the SCVs exhibited decreased intracellular bacterial number in RAW264.7 macrophages and were highly attenuated for virulence in zebrafish. Considering the outer membrane proteins (OMPs) are directly involved in bacterial interaction with the external surroundings, we investigated the roles of OMPs in the antipredator fitness behaviors of *A. hydrophila*. A total of 38 differentially expressed proteins were identified in the SCVs by quantitative proteomics. Among them, three lipoproteins including SurA, Slp, and LpoB, and a serine/threonine protein kinase (Stpk) were evidenced to be associated with environmental adaptation of the SCVs. Also, the three lipoproteins were involved in attenuated virulence of SCVs through the proinflammatory immune response mediated by TLR2. This study provides an important contribution to the understanding of the defensive traits of *A. hydrophila* against protistan predators.

## Introduction

*Aeromonas hydrophila* is a gram-negative bacterium that is ubiquitously found in various aquatic environments, including rivers, ponds, groundwater, seawater, wastewater and sewage (Janda and Abbott, 2010). *A. hydrophila* is responsible for outbreaks of motile aeromonad septicemia (MAS), which leads to huge economic losses in the global aquaculture industry (Galindo et al., 2006). In addition to causing fish disease, *A. hydrophila* is also associated with various severe diseases in other cold- and warm-blooded animals, including humans (Janda and Abbott, 2010). The survival and pathogenicity of *A. hydrophila* is influenced by multiple factors, such as adhesins (Tomás, 2012), proteases (Pemberton et al., 1997; Tomás, 2012), and the ability to form biofilms (Talagrand-Reboul et al., 2017). However, pathogens do not exist in isolation. The interaction among organisms is recognized as a major influencing factor with respect to the survival and evolution of bacteria in the environment (Holt and Roy, 2007; Borer et al., 2009). As an integral part of the environmental microbial community, bacterial pathogens also form the base of many food webs and are constantly confronted with strong predation pressure by heterotrophic bacterivorous protists (Gasol et al., 2002; Li et al., 2011).

Protists are eukaryotic unicellular microorganisms that are ubiquitous in almost all environments. Grazing by protists is regarded as a major cause of bacterial mortality in most soil, freshwater and marine ecosystems (Fenchel, 1987). Protists have been suggested to tightly control bacterial populations, but also function as protective reservoirs (Brown and Barker, 1999). Studies have demonstrated that certain protists can protect bacterial pathogens from various environmental countercurrents and provide an ideal environment for bacterial replication (Barker and Brown, 1994; Matz and Jürgens, 2003). *Mycobacterium avium* isolates that are able to survive within free-living amoeba are protected from the adverse effects of antimicrobials and results in increased virulence (Cirillo et al., 1997; Miltner and Bermudez, 2000). *Salmonella enteritidis* isolates which survived *Tetrahymena* grazing had a strong resistance to calcium hypochlorite and showed a enhanced acid-resistance ability (Brandl et al., 2005; Rehfuss et al., 2011). *Legionella pneumophila* residing in pellets expelled by *Tetrahymena tropicalis* exhibited an increase in gentamicin resistance and survival in nutrient-poor environments (Koubar et al., 2011).

In addition to the defensive traits displayed by bacteria harbored inside protists or pellets, the development of predation resistance is another driving force for bacterial evolution that contributes to the diversification of bacteria. Defensive strategies of bacteria that provide protection from protistan predation could have evolved in response to grazing mortality, such as size-reduction, microcolony and biofilm formation, toxin production, and alterations in motility, cell morphology and outer membrane protein structure (Weekers et al., 1993; Matz and Kjelleberg, 2005; Pernthaler, 2005). Moreover, many of these traits used to survive protistan grazing are essential prerequisites for the environmental persistence of bacterial pathogens, which might have also resulted in enhanced environmental adaptability and pathogenicity (Matz et al., 2004, 2005; Adiba et al., 2010). In contrast, it has previously been reported that bacterial pathogens that have undergone protistan predation pressure for prolonged periods, their outside-host defensive and adaptive mechanisms can have a fitness trade-off with virulence related characteristics, resulting in a decrease in virulence and pathogenicity (Friman et al., 2009; Mikonranta et al., 2012; Zhang et al., 2014a). Nevertheless, it remains unclear whether protistan predation will have an effect on the environmental adaptation and pathogenicity of *A. hydrophila*.

*Tetrahymena* is a primary bacterivorous protist that lives in the same habitat as *A. hydrophila* (Pang et al., 2012). In this study, to gain a better understanding of the defense mechanisms of *A. hydrophila* against protistan predation, we investigated the morphological and adaptive effects that *T. thermophila* predation has on the Chinese epidemic strain *A. hydrophila* NJ-35. In addition, we analyzed the molecular mechanisms involved in defense strategies and discuss the potential role of *T. thermophila* in the persistence and adaption of *A. hydrophila* in aquatic environments.

## Materials and methods

### Strains, cell lines, and culture conditions

The *A. hydrophila* strain NJ-35 was isolated from diseased cultured crucian carp in Nanjing, China in 2010 (Pang et al., 2012). The complete genome sequence of NJ-35 has been published in GenBank (accession number CP006870). The *Escherichia coli* strain BL21, which carried the kanamycin-resistant plasmid pET-28a (+), was stored in our laboratory. *A. hydrophila* and *E. coli* were routinely cultured in Luria Bertani broth (LB) (Difco/Becton Dickinson) at 28 and 37°C, respectively.

*T. thermophila* SB210 (Eisen et al., 2006) was obtained from Dr. Miao Wei, Institute of Hydrobiology, Chinese Academy of Sciences. The whole genome sequence of *T. thermophila* SB210 has been deposited in GenBank (accession number GCA_000261185.1). *T. thermophila* SB210 was grown axenically in SPP medium (containing 2% proteose peptone, 0.1% yeast extract, 0.2% glucose, and 0.003% EDTA-Fe) at 28°C (Pang et al., 2012).

The lytic bacteriophage G65 used to infect *A. hydrophila* NJ-35 was isolated from a contaminated river in Nanjing, China, in 2014. G65 is a T4-like bacteriophage belonging to the family *Myoviridae*.

RAW264.7 cells (ATCC) and HEp-2 cells (ATCC) were maintained in Dulbecco's modified Eagle medium (DMEM) with high glucose (Gibco, Invitrogen Corp., Carlsbad, CA) supplemented with 10% (vol/vol) fetal bovine serum (FBS) (Gibco) at 37°C with 5% CO_2_. All reagents used in this study were supplied by Sigma (St. Louis, MO, USA) unless otherwise indicated.

### Passaging of *A. hydrophila* in the presence of *T. thermophila*

*A. hydrophila* passaging was performed as previously described (Örmälä-Odegrip et al., 2015) with some modifications. The medium used to co-culture *A. hydrophila* NJ-35 with *T. thermophila* contained LB nutrients at a 5% concentration, with TBSS used as the solvent (0.5 g NaCl, 0.5 g Tryptone and 0.25 g Yeast Extract dissolved in 1 liter of TBSS; TBSS, 2 mM KCl, 1 mM CaCl_2_, 0.5 mM MgCl_2_, and 1 mM Tris, pH 7.0). The experiment was initiated from a single ancestral colony of *A. hydrophila* NJ-35. The ancestor strain was cultured alone or co-cultured with *T. thermophila* SB210. And the ratio of predator to prey set up was 1:5,000, as described by Pang et al. (2012). Each treatment was replicated in triplicate in 50-ml glass vials containing 12 ml of co-culture medium. Vials were kept at 28°C without shaking. Every 48 h, 20% of each culture was transferred into a new vial containing fresh co-culture medium. During each transfer, a 0.5 ml subsample was mixed with 0.5 ml of 50% glycerol and kept at −70°C for the preservation of the bacteria and the lysis of *Tetrahymena*. The semi-continuous subculture lasted for 4 weeks. Afterwards, the lysate was diluted and put onto the LB agar plates to isolate the single colonies that were exposed or unexposed to *Tetrahymena*.

### Bacterial growth curves

A single colony from each *A. hydrophila* strain was cultured overnight 28°C and the OD_600_ values were regulated to 0.5 with fresh LB medium. The cultures were diluted 1:100 into a flask containing 20 ml of LB medium. Then, the flasks were incubated with shaking for 16 h at 180 rpm at 28°C. Every 1 h, the OD_600_ was monitored using a spectrophotometer (BIO-RAD, USA). The growth experiments for each strain were repeated three times. And data were collected from three independent experiments.

### Biofilm formation assay

Biofilm formation was measured by crystal violet staining as previously described (O'Toole et al., 1999). *A. hydrophila* strains were cultured in LB medium to an OD_600_ of 0.6–0.8 and then normalized to 0.1. The normalized cell suspensions were inoculated into 200 μl of fresh LB (1:100 dilution) and placed into a 96-well plate, followed by incubation at 28°C for 24 h without shaking. Cultures of each strain were replicated in eight different wells. Fresh LB medium was added to the wells as a blank control. Next, the medium was aspirated and the plate was washed three times with sterile PBS to remove any unbound cells. After washing, each well was fixed with 200 μl of 99% (vol/vol) methanol for 15 min and allowed to air dry at room temperature. After drying, 200 μl of a 1% crystal violet solution was added to each well and incubated for 10 minutes at room temperature. The plate was then rinsed with distilled water to remove any unbound crystal violet. The bound crystal violet was dissolved from each well using 200 μl of 95% ethanol for 10 min. The absorbance at 595 nm (OD_595_) was measured using a micro-plate reader (Tecan, Switzerland). The assay was performed in three independent experiments.

### Motility assay

The swimming motility and swarming motility assays were performed using 0.3% and 0.5% agar plates, respectively, as previously described (Khajanchi et al., 2012). *A. hydrophila* strains grown to log phase were adjusted to an OD_600_ of 1.0. One microliter of each suspension was stabbed into the LB semi-fluid agar plates. Each strain was replicated three times. After incubating for 48 h at 28°C, motility was assessed by measuring the distance of bacterial migration from the inoculation point, and photographs were taken using a gel imaging system (Bio-Rad, USA). The assay was performed in three independent experiments.

### Adhesion assay

The adhesion assay was performed using HEp-2 cells as previously described (Tan et al., 2014). The HEp-2 cells were grown in 24-well plates to obtain monolayer cells. *A. hydrophila* strains grown to exponential phase were harvested in fresh serum-free MEM and then transferred to each well to infect HEp-2 cells with a multiplicity of infection (MOI) of 1:1. Bacteria added to the wells without HEp-2 cells served as a control. Each strain was replicated in four different wells. The plate was centrifuged at 800 × g for 10 min and incubated at 37°C with 5% CO_2_ for 2 h to allow for cell adhesion. The non-adherent bacteria were removed by washing three times with PBS. After washing, the cells were lysed with 1 ml of 0.1% Triton X-100 (vol/vol) for 10 min. The adhered bacteria were quantified using LB agar plates. The relative adhesion was calculated as the CFU of adhered cells divided by that of the bacteria cultured alone. The assay was performed in three independent experiments.

### Susceptibility to phage

The bacterial susceptibility to infection by phages was evaluated by detecting the phage titers. *A. hydrophila* strains cultured for 4 h were adjusted to a density of 1 × 10^8^ CFU/ml. One hundred microliters of each bacterial suspension was mixed with aliquot phage G65 (1 × 10^8^ PFU/ml) and incubated for 15 min at 28°C. Then the mixture was added to 2.8 ml of LB medium and incubated with shaking for 4 h at 180 rpm at 28°C. Biological triplicates were performed for each strain. After incubation, the supernatant of infected cultures were collected by centrifugation and filtered through a 0.22 μm filter. The phage titer in the supernatants was determined by counting the plaques using a double layer plaque assay (Cormier and Janes, 2014). The assay was performed in three independent experiments.

### Determination of adsorption curve by phage

*A. hydrophila* strains grown to log phase were adjusted to an OD_600_ of 0.2 with LB media. Next, 1 ml of phage G65 (2.0 × 10^5^ PFU/ml) was added into 9 ml of the bacterial suspension, which was then incubated at 28°C for 30 min. Phage mixed with LB media without bacteria served as a control. At a regular 2.5 min intervals, 100 μl from each co-culture was placed into 950 μl of 4°C LB. The mixtures were put under vigorous vortex for 10 s and then centrifuged at 10,000 g for 10 min at 4°C. The titer of the free phage in the supernatants was determined by a double layer plaque assay (Cormier and Janes, 2014). Each measurement was repeated in quadruplicate. The adsorption curve was drawn with the time as abscissa and the percentage of unabsorbed free phage as the ordinate. The assay was performed in three independent experiments.

### Anti-bacterial competition assay

The *E. coli* inhibition assay was performed as previously described with some modifications (Decoin et al., 2015; Chatzidaki-Livanis et al., 2016). Both *A. hydrophila* and *E. coli* strains grown to an OD_600_ of 1.0 were concentrated 10 times and mixed at a ratio of 1:1. A total of 25 μl of the above mixtures were spotted onto 0.22 μm sterile filters fixed on LB agar plates. *E. coli* BL21 cells mixed with equal volume of LB media was used as a control. After incubation at 28°C for 3 h, the spots were suspended in LB media and 10-fold serially diluted. The survival *E. coli* in the dilutions were determined using a viable cell count on LB agar plates containing kanamycin (50 μg/ml). The ability of *A. hydrophila* strains to compete against *E. coli* was expressed as the CFU of viable *E. coli* cells after *A. hydrophila* antagonism. The assay was performed in triplicate with three independent experiments.

### Antioxidant stress assay

The antioxidant stress tests were performed by detecting the viability of *A. hydrophila* strains with H_2_O_2_ exposures. Exponential-phase cultures were normalized to an OD_600_ of 0.1 before being treated with 1 ml of 2 mM H_2_O_2_ for 50 min at 28°C. After treatment, the oxidation was terminated with 2,000 U of catalase for 10 min. The CFU of viable cells post-treatment were scored using a viable cell count. The resistance levels against H_2_O_2_ were expressed as the number of survival *A. hydrophila* post treatment. The assay was performed in triplicate with three independent experiments.

### Bacterial survival in RAW264.7 macrophage cells

RAW264.7 macrophages were grown in DMEM containing 10% FBS in 24-well tissue plates at a concentration of 4 × 10^5^ cells/well. *A. hydrophila* strains cultured to exponential phase were collected in fresh serum-free MEM. The macrophage cells were infected by bacterial suspension with a MOI of 1:1 for 30 or 60 min. Then extracellular bacteria were inactivated by culturing with 1 ml 100 μg/ml gentamicin sulfate for 40 min. After incubation, infected cells were washed three times with sterile PBS, followed by the addition of 1 ml 0.1% (vol/vol) Triton X-100 for 10 min to fully lyse the macrophages and release intracellular bacteria. The number of intracellular bacteria were quantified using LB agar plates. The assay was performed in quadruplicate with three independent experiments.

### LDH cytotoxicity assay

Cytotoxicity of RAW264.7 macrophage cells induced by *A. hydrophila* was evaluated by measuring the release of lactate dehydrogenase (LDH) with a CytoTox 96 nonradioactive cytotoxicity assay (Promega). The assay was performed according to the manufacturer's instructions. Briefly, RAW264.7 cells grown in 96-well plate were infected with aliquots of *A. hydrophila* cells (MOI of 1.0). The plate was centrifuged at 800 g for 10 min and then incubated for 3 h at 37°C with 5% CO_2_. The LDH released by lysis of cells with 1% (vol/vol) Triton X-100 was defined as cell maximum release. And the LDH released by uninfected cells and bacteria alone was designated spontaneous release. The release of LDH was measured at OD_490_. Cytotoxicity was calculated as follows: % cytotoxicity (test LDH release—cell spontaneous release—bacteria spontaneous release)/(cell maximal release—cell spontaneous release). The assay was performed in three independent experiments.

### Protease activity

Protease activities were performed as previously described (Swift et al., 1999). *A. hydrophila* strains grown for 18 h were adjusted to an OD_600_ of 2.0. Cells were removed from the culture by centrifugation, and 250 μl aliquots of supernatants were added to 250 μl of 0.5% (wt/vol) azocasein in 50 mM Tris-HCl (pH 8.0) and incubated at 37 °C for 2 h. The proteins were precipitated by the addition of 500 μl of ice-cold 10% (wt/vol) TCA followed by incubation on ice for 30 min. After the removal of precipitated protein by centrifugation, 500 μl of the supernatants were taken out and added to an equal volume of 1 M NaOH. Azodye released by the action of proteases in supernatants was measured at OD_440_. The assay was performed in quadruplicate with three independent experiments.

### Determinations of LD_50_ in zebrafish

The animal experiment was carried out in accordance with the animal welfare standards and complied with the guidelines of the Animal Welfare Council of China and was approved by the Ethical Committee for Animal Experiments of Nanjing Agricultural University, China. The virulence of *A. hydrophila* strains were assessed by the 50% lethal dose (LD_50_) values in a zebrafish model (Pang et al., 2012). The zebrafish used in this study were bought from the Pearl River Fishery Research Institute, Chinese Academic of Fishery Science. Logarithmic phase bacteria were washed three times with sterile PBS and serially tenfold diluted to densities of 5 × 10^2^ to 5 × 10^7^ CFU/ml. For each *A. hydrophila* strain, seven groups of 11 zebrafish were intraperitoneally injected with 20 μl of bacterial suspensions containing 10^1^ to 10^7^ CFU in PBS. An additional 11 zebrafish that were injected intraperitoneally with PBS served as controls. Mortality was recorded for 7 days. The assay was performed in four independent experiments and the LD_50_ values were calculated by the method of Reed and Muench (1938).

### Mass spectrometry analysis

A mass spectrometry analysis was used to study the adaptation mechanisms of *A. hydrophila* NJ-35 in response to protistan predation. The outer membrane proteins (OMPs) of the strains that were exposed or unexposed to *Tetrahymena* were extracted and analyzed through a mass spectrometry analysis. Log-phase bacteria (three biological replicates) were washed once and resuspended in ice-cold 0.02 mol/L Tris-HCl (pH 7.5). The bacterial suspensions were ruptured by sonication at 4°C and then centrifuged (7,000 g, 10 min, 4°C) to collect the supernatants. The supernatants were ultracentrifuged at 190,000 g for 30 min at 4°C to obtain pellets. The pellets were resuspended in 20 ml 0.5% (w/v) SLS (Sodium dodecylphosphate) and were stored at 4°C overnight. Next, the solution was ultracentrifuged (190,000 g, 30 min, 4°C) to precipitate the pellets (OMPs), which were then resuspended in ddH_2_O_2_ and preserved at −20°C. The OMPs were analyzed quantitatively using a label free method of mass spectrometry.

### Quantitative reverse transcription-PCR (qRT-PCR)

Based on the proteomics results, we randomly selected 14 differentially expressed proteins from 38 proteins (above 1/3) and measured the mRNA expression levels by qRT-PCR. RNA was extracted from logarithmic-phase bacteria using an E.Z.N.A. bacterial RNA kit (Omega, USA). The mRNA transcription levels of the 14 genes were examined individually using a One Step qRT-PCR SYBR Green kit (Vazyme Biotech) in an ABI PRISM 7300 Fast Real-time PCR machine. For each sample, the acquired cycle threshold (CT) was normalized to the CT of the internal housekeeping gene *recA*. The fold-change of mRNA expression levels were calculated using the 2^−ΔΔCT^ method as previously described (Livak and Schmittgen, 2001). The primers used are described in Table S1.

### Roles of *surA, slp, lpoB*, and *stpk* in the adaptability of *A. hydrophila*

Four genes encoding differentially expressed proteins, including *surA, slp, lpoB*, and *stpk*, were selected to determine their relationship with the adaptive traits of *A. hydrophila*. For the *stpk* shown to be downregulated, we constructed the *stpk* overexpressing strain using the pMMB207 shuttle plasmid. The complete *stpk* gene and its putative promoter and terminator regions were amplified and ligated into the pMMB207 vector. The recombinant plasmid *stpk*-pMMB207 was transformed into *A. hydrophila* by bacterial conjugation, thus generating the *stpk*-upregulated strains. In addition, the pMMB207 empty plasmid was also transformed into *A. hydrophila* to serve as controls. For the other three genes shown to be upregulated, the gene-deletion mutants were respectively constructed in *A. hydrophila* via homologous recombination using the suicide plasmid pYAK1 as previously described (Pang et al., 2016). Briefly, two flanking regions of the target gene were amplified and ligated by PCR. The fusion fragment was inserted into pYAK1 and transformed into *E. coli* SM10. The recombination vector from the donor strain *E. coli* SM10 (chloramphenicol resistant, Cm^r^) was conjugated into the recipient *A. hydrophila* strain (ampicillin resistant, Amp^r^). Strains having undergone allelic exchange were selected with LB agar plates containing 100 μg/ml Amp and 34 μg/ml Cm. The positive colonies were cultured in LB media without sodium chloride for 12h and then transferred to LB agar plates containing 20% sucrose to generate the deletion mutants. The suspected mutants were verified by PCR. The primers used for mutants construction are listed in Table S2.

Biofilm formation, cellular adhesion ability, antioxidant stress and virulence were evaluated as described above. Bacterial resistance to predation was assessed by measuring the relative survival of bacteria after co-culture with *T. thermophila* (Pang et al., 2016). *A. hydrophila* (1 × 10^9^ CFU/ml) and *T. thermophila* SB210 (2 × 10^5^ cells/ml) were mixed at a volume ratio of 1:1 in TBSS. Then, 100 μl of the mixture was added to each well of a 96-well plate. The *A. hydrophila* and *T. thermophila* suspensions were mixed with an equal volume of TBSS respectively to serve as controls, and TBSS alone served as a blank. Each group was performed in four different wells. The plate was cultured at 28°C for 12 h and OD_450_ was measured. The relative survival of bacteria was expressed as the OD_450_ value of bacteria co-cultured with *T. thermophila* divided by that of bacteria grown alone at 12 h. Data were collected in four independent assays.

### Macrophage infections and stimulations

RAW264.7 macrophages seeded into 6-well plates were infected with *A. hydrophila* at an MOI of 1:1. Uninfected RAW264.7 macrophages served as controls. Cells were incubated for 1 h at 37°C and washed three times before adding antibiotics. To measure the Toll-like receptor 2 (TLR2) and cytokine expression of the infected cells, cells were sampled at 3 h after the addition of antibiotics. Then total RNA was isolated from the macrophages using an E.Z.N.A. total RNA kit I (Omega, USA). The mRNA levels were measured using two-step relative qRT-PCR in an ABI Step One Plus qPCR machine. The β-actin housekeeping gene was amplified as an internal control. The sequences of the primers for TLR2, tumor necrosis factor alpha (TNF-α), interleukin-1beta (IL-1β), IL-6, and β-actin are listed in Table S3. The comparative cycle threshold (2^−ΔΔCT^) method was used to analyze the mRNA levels (Livak and Schmittgen, 2001). The assay was performed in triplicate with three independent experiments.

### Statistical analyses

Data were collected and analyzed using GraphPad Prism version 5 software. Tukey's multiple comparisons were performed using one-way analysis of variance (ANOVA) with 95% confidence intervals. The biofilm formation, adhesion, anti-protistan predation and antioxidant stress levels of *A. hydrophila* parental strain and the derived mutants were analyzed using *t*-test. *P*-values < 0.05 were considered as statistically significant.

## Results

### Phenotypic features of *A. hydrophila* clones co-cultured with *T. thermophila*

**Figure 1:**
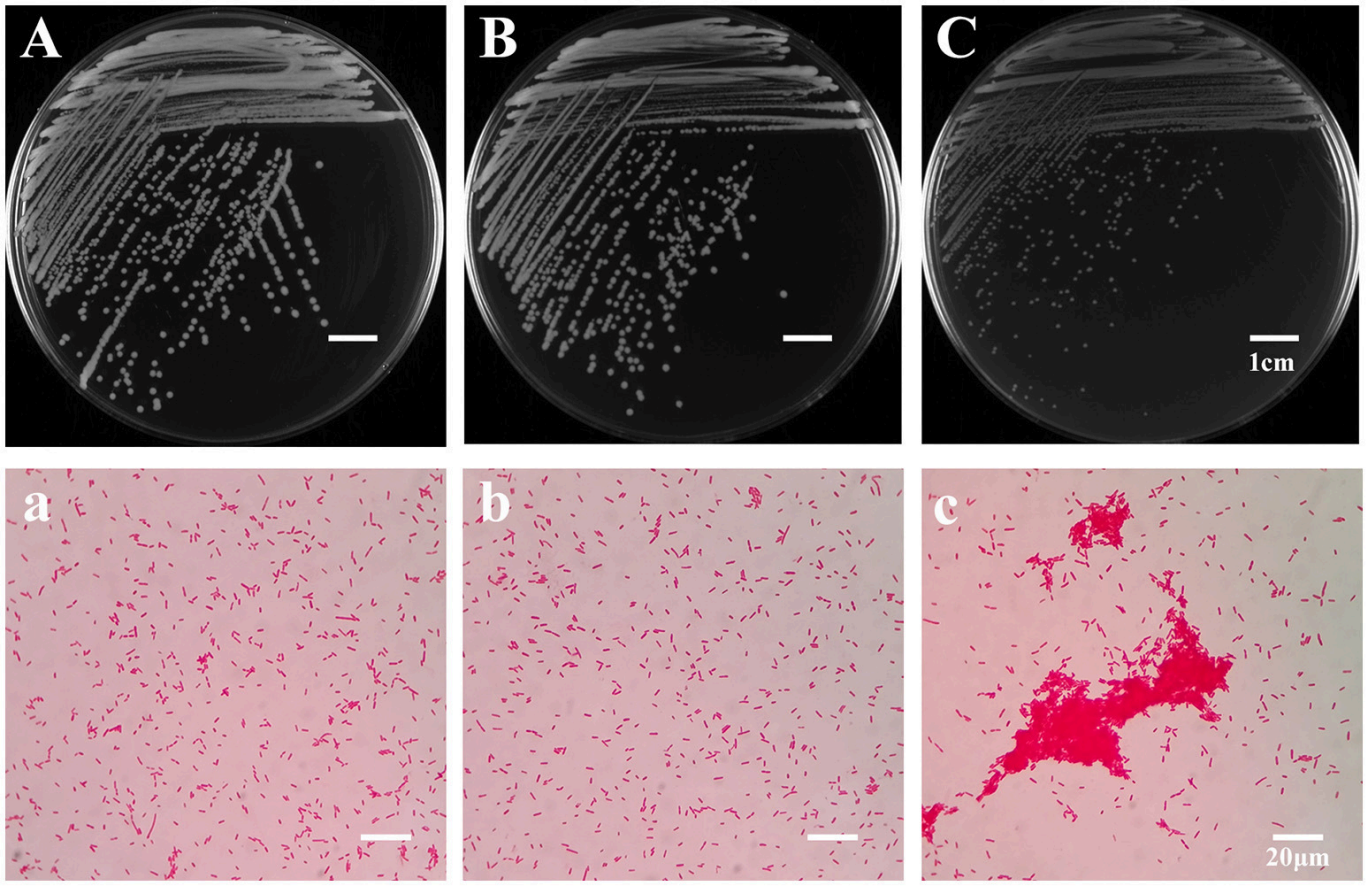
Colony and microscopic morphology of the ancestor, B strains (non-*Tetrahymena*-exposed) and SCV strains (*Tetrahymena*-exposed). The colony morphology of the ancestor **(A)**, B1 **(B)**, and SCV1 **(C)** strains on the LB plate were observed after culture for 20 h. The microscopic morphology of the ancestor **(a)**, B1 **(b)**, and SCV1 **(c)** strains were observed at 100 × magnification after Gram staining. The bar represents 1 cm in **(A–C)** and 20 μm in **(a–c)**.

To explore the evolutionary mechanisms of *A. hydrophila* under the grazing pressure of *T. thermophila*, a co-culture experiment lasting 4 weeks between *A. hydrophila* NJ-35 and *T. thermophila* SB210 was carried out. After passaging, bacterial colonies isolated from the treatments with or without *T. thermophila* were defined as BT strains and B strains respectively. BT strains exhibited two types of colony morphologies when grown on LB plates. The first (data not shown) was yellowish white with an opaque colony phenotype that was similar to the ancestor (Figure 1A) and B strains (Figure 1B), while the second accounted for 78.62% were small colony variants (SCVs), which have an off-white and transparent phenotype on LB plates (Figure 1C). Under the light microscope, the SCV cells (Figure 1c) were of normal size and shape but displayed aggregation phenomenon compared to the ancestor (Figure 1a) and B strains (Figure 1b). No obvious difference in colony morphology was observed between the B strains and the ancestor strain. Then we randomly selected six SCV strains (SCV1 thru SCV6) and three B strains (B1 thru B3) for the examination of adaptive traits, such as biofilm formation, motility, adhesion and environmental stress.

### Growth and biofilm forming ability

**Figure 2:**
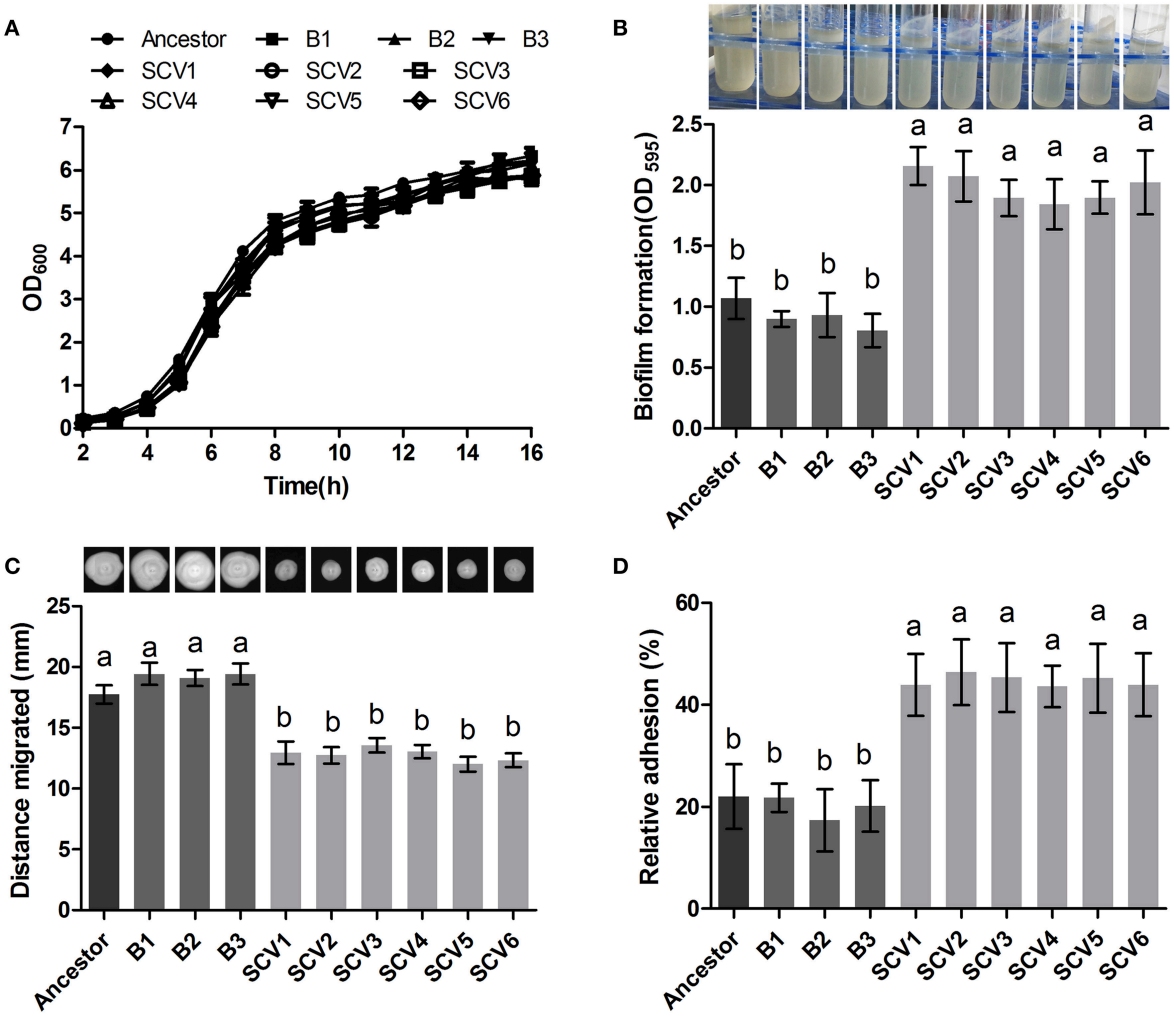
Growth curve, biofilm formation, motility and cell adhesion of the ancestor, B (non-*Tetrahymena*-exposed) strains and SCV strains (*Tetrahymena*-exposed). **(A)** Growth curves of the ancestor, B strains and SCV strains. The strains were grown in LB medium. **(B)** Biofilm formation was measured with crystal violet staining using 96-well plates, and it was expressed as the values of OD_595._ The tubes showed that lots of biofilm was formed on the tube wall after SCVs culture overnight with a shaker at 28°C. **(C)** Swimming ability was observed after culturing at 28°C for 48 h on 0.3% LB agar plates. The swimming distance was measured from inoculation point. **(D)** The relative adhesion was calculated by dividing the number of colony-forming units (CFU) of adhered bacteria by the number of CFU of the inoculum. Data are presented as the mean ± SD of three independent experiments, with each experiment being consisting of three replicates. Different lowercase letters (a, b) indicate significant differences (*P* < 0.05) among different bacterial strains.

The ancestor, B and SCV strains showed no significant difference (*P* > 0.05) in bacterial growth when cultured in LB medium for 16 h (Figure 2A). However, biofilm-formation ability of the SCV strains was significantly increased (by 127.86%) compared to the B strains (*P* < 0.001) (Figure 2B). And for the SCV strains, lots of biofilm could also be observed on the walls of the culture test tubes after cultured overnight in a shaker (Figure 2B). No obvious difference in biofilm production was detected between the B strains and the ancestor strain.

### Swimming and swarming motilities

The swimming motility of *A. hydrophila* was measured by examining distance migrated from the inoculation center on 0.3% LB agar plates. Migration diameters of 17.73 ± 0.75, 19.32 ± 0.72, and 12.77 ± 0.77 mm were measured for the ancestor, B strains and SCV strains, respectively (Figure 2C), indicating that swimming motility of SCVs were significantly decreased compared with the ancestor (*P* < 0.05) and B strains (*P* < 0.05). Similarly, the swarming motility presented a similar trend as was observed for the swimming motility (data not shown).

### Adhesion to HEp-2 cells

The adherence capacities of the ancestor strain, B strains and SCV strains were tested using HEp-2 cells. The relative adhesion rate of the SCVs (44.73 ± 5.239 %) was significantly increased compared to both the B strains (19.77 ± 4.633 %) (*P* < 0.01) and the ancestor strain (22.00 ± 6.307 %) (*P* < 0.01) (Figure 2D). A similar adherence ability was observed when comparing the B strains and the ancestor strain.

### Susceptibility to phage

**Figure 3:**
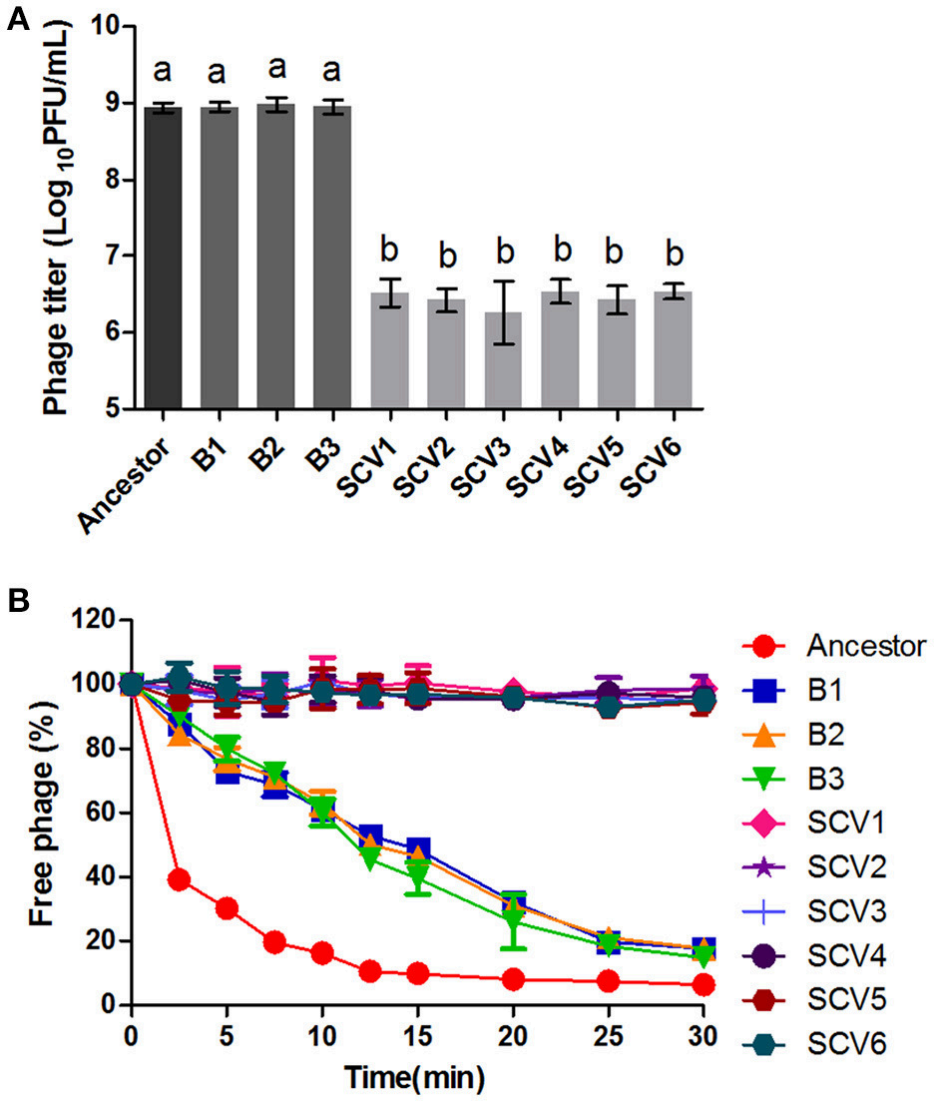
Susceptibility of the ancestor, B strains (non-*Tetrahymena*-exposed) and SCV strains (*Tetrahymena*-exposed) to phage. **(A)** Relative bacteriophage titer was defined as the plaque-forming units (PFU) of phage after coculture with the bacterial strains for 4 h. **(B)** The adsorption curves of phage. The titer of the free phage was determined after co-incubation of phage G65 with the bacterial strains at a regular interval of 2.5 min. The phages cultured alone served as control. The adsorption curve was drawn with time as abscissa and the percentage of un-adsorbed free phage as ordinate. Data are presented as the mean ± SD of three independent experiments, with each experiment being consisting of three replicates. Different lowercase letters (a, b) indicate significant differences (*P* < 0.05) among different bacterial strains.

Susceptibility to phage was defined as the titers of the phage G65 observed after co-culturing with the ancestor strain, B strains, or SCV strains. As shown in Figure 3A, almost no proliferation of phage was observed when it was co-cultured with the SCVs, with a biomass of (3.08 ± 1.17) × 10^6^ PFU/ml. In contrast, the biomass of phage co-cultured with the B strains and the ancestor strain were (8.75 ± 1.24) × 10^8^ and (9.29 ± 1.65) × 10^8^ PFU/ml, respectively. To compare the adsorption efficiency of phage G65 to the ancestor, B and SCV strains, adsorption curves were determined. As shown in Figure 3B, phage adsorption to the ancestor strain was approximately 60% within 2.5 min, and more than 90% after 15 min. Phage adsorption to the B strains was slightly lower than that of the ancestor strain at the corresponding times, and achieved 80% adsorption at 25 min. However, no phage adsorption to the SCVs was detected within 30 min.

### Anti-bacterial competition ability

**Figure 4:**
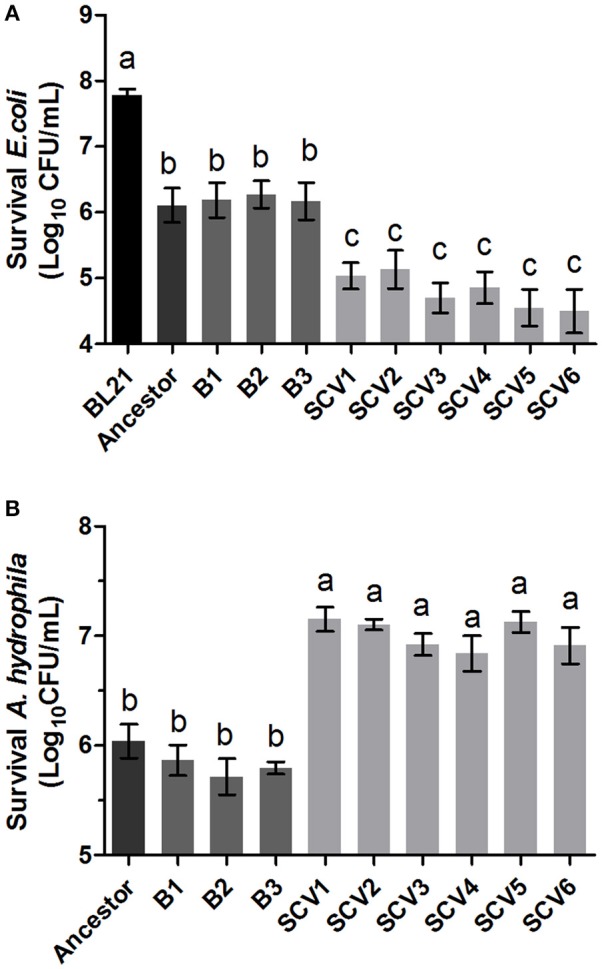
Competition ability and H_2_O_2_ resistance levels of the ancestor, B strains (non-*Tetrahymena*-exposed) and SCV strains (*Tetrahymena*-exposed). **(A)** The competition capability of *A. hydrophila* strains against *E. coli* BL21 was defined as the amount of survival *E. coli* after antagonism. **(B)** The H_2_O_2_ resistance levels of the ancestor, B strains and SCV strains were expressed as the CFU of the viable *A. hydrophila* after treatment with H_2_O_2_. Data are presented as the mean ± SD of three independent experiments, with each experiment being comprised of four individual measurements. Different lowercase letters (a, b, c) indicate significant differences (*P* < 0.05) among different bacterial strains.

The ability of *A. hydrophila* to antagonize *E. coli* BL21 was tested by co-culturing with the ancestor strain, B strains and SCV strains. The *E. coli* BL21 strain contained plasmid pET-28a (+), which conferred resistance to kanamycin to allow for the selection of viable *E. coli* BL21 cells after antagonism. Competitions were performed for all *A. hydrophila* strains against *E. coli* BL21 (Figure 4A). Co-culturing of *E. coli* BL21 with the SCVs brought about a one-log reduction in CFU compared to *E. coli* that was co-cultured with either the ancestor strain or the B strains (*P* < 0.001).

### Resistance to oxidative stress

The resistance levels to oxidative stress in the ancestor strain, B strains and SCV strains were determined by treating each strain with H_2_O_2_. As shown in Figure 4B, the B strains did not show a significant alteration in H_2_O_2_ (2 mM) resistance levels compared to the ancestor strain. However, the H_2_O_2_ resistance level of SCVs was approximately ten-fold greater than that of the B strains (*P* < 0.001).

### Effect on RAW264.7 macrophage cells

**Figure 5:**
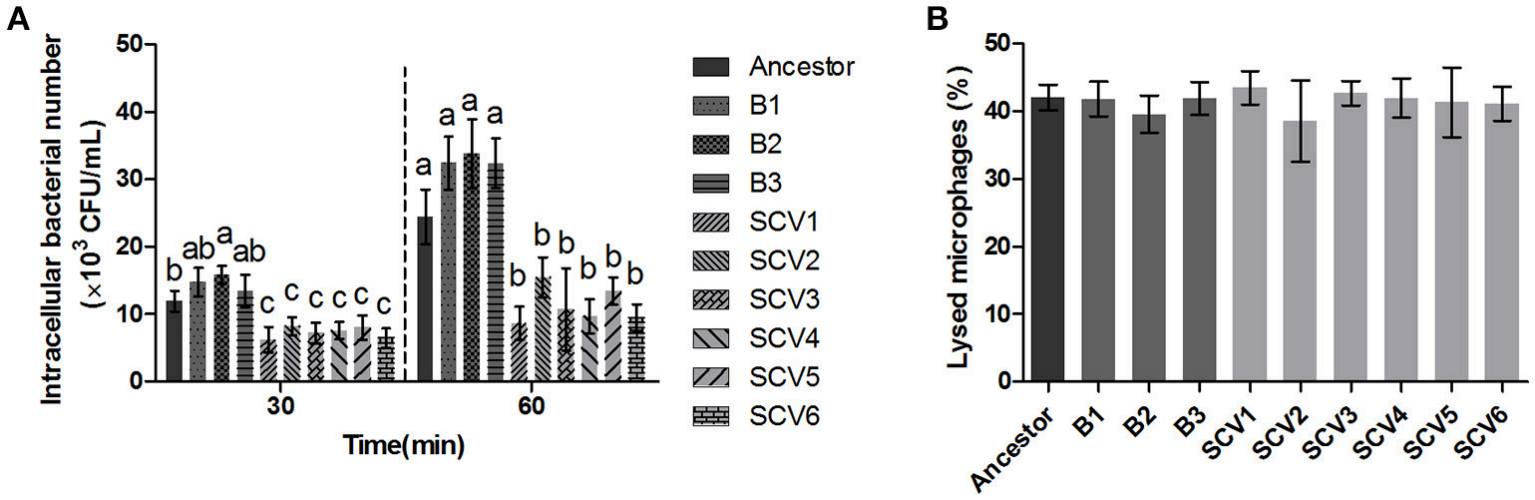
Effect of the ancestor, B strains (non-*Tetrahymena*-exposed) and SCV strains (*Tetrahymena*-exposed) on RAW264.7 Cells. **(A)** The intracellular bacteria of *A. hydrophila* strains was determined as the number of bacteria within macrophages after 30 and 60 min post-infection. The multiple comparisons among the strains have been made at time 30 and time 60, respectively. Different lowercase letters (a, b, c) indicate significant differences (*P* < 0.05) among different bacterial strains at the same time point. **(B)** Effect of bacterial cell-mediated cytotoxicity was determined by measuring the LDH release into the supernatant of RAW264.7 macrophages post-infection for 3 h. Data are presented as the mean ± SD of three independent experiments, with each experiment being comprised of three individual measurements.

The bacterial number of *A. hydrophila* strains within macrophages was determined after 30 and 60 min post-infection. As shown in Figure 5A, the number of intracellular SCVs were significantly lower than that of the ancestor and B strains both at time 60 and time 30. Further, the results of LDH assay demonstrated that the cytotoxic effect of SCVs on RAW 264.7 cells was similar to that of B strains (Figure 5B), suggesting the reduced number of SCVs in macrophages was not the result of the bacterial toxicity.

### Protease activity

**Figure 6:**
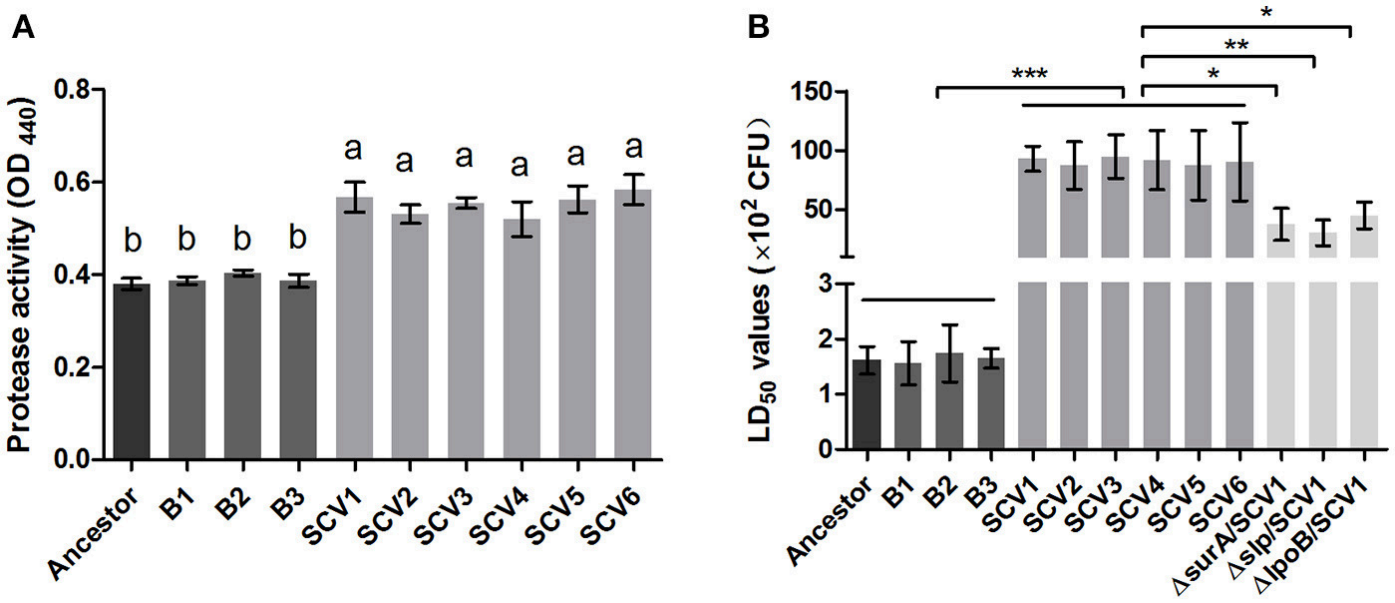
Protease activity and LD50 determination. **(A)** The protease activity in the culture supernatants of the ancestor, B strains (non-*Tetrahymena-*exposed) and SCV strains (*Tetrahymena-*exposed) was detected using azocasein as protease substrate and measured at OD_440_. Data are presented as the mean ± SD of three independent experiments, with each experiment being comprised of four individual measurements. Different lowercase letters (a, b) indicate significant differences (*P* < 0.05) among different bacterial strains. **(B)** The LD_50_ values of the ancestor, B strains, SCV strains, and *surA, slp, lpoB* gene-deletion mutants in SCV1 were calculated using a zebrafish model. Data are presented as the mean ± SD of four independent experiments. ^*^P < 0.05, ^**^0.001 < *P* < 0.01, or ^***^P < 0.001 indicates significant difference among different bacterial strains.

For quantifying the protease activity in *A. hydrophila*, assays using culture supernatants from the ancestor strain, B strains and SCV strains were subjected to azocasein. The results in Figure 6A showed that analysis of supernatants revealed an increased protease production in the SCVs (0.55 ± 0.033) compared with both the ancestor strain (0.38 ± 0.012) (*P* < 0.001) and the B strains (0.39 ± 0.012) (*P* < 0.001).

### LD_50_ determinations in zebrafish

To determine whether the predation of *T. thermophila* affected the virulence of *A. hydrophila*, the pathogenicity of the ancestor strain, B strains and SCV strains was investigated using a zebrafish model. The LD_50_ values of the SCV strains were approximately 50-fold higher than that of the ancestor strain and the B strains (Figure 6B), indicating that the SCV strains were highly attenuated for virulence compared with the ancestor and B strains.

### Mass spectrometry analysis of membrane proteins

**Figure 7:**
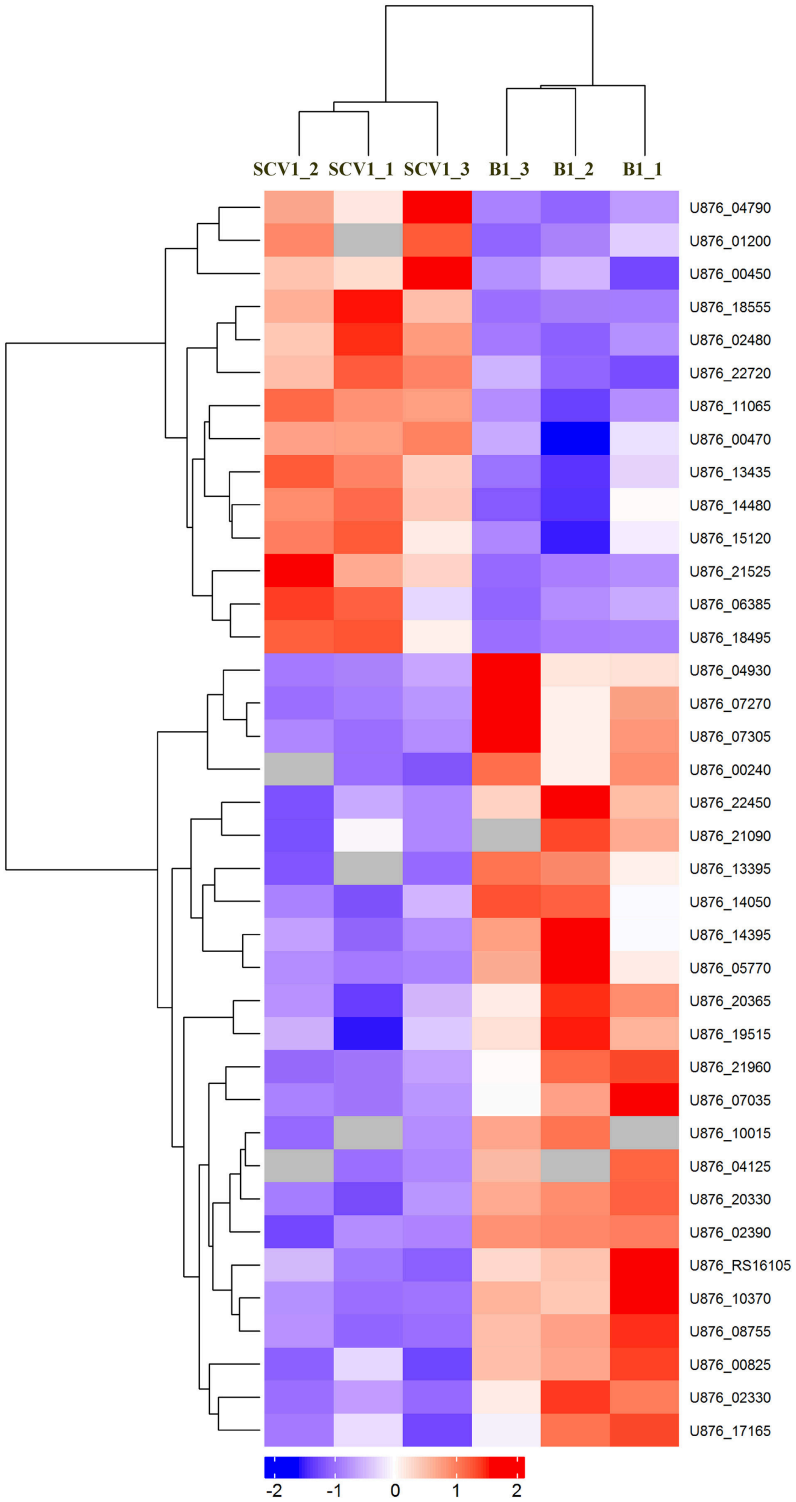
Heatmap of differentially expressed proteins between the B1 strain (non-*Tetrahymena*-exposed) and SCV1 strain (*Tetrahymena*-exposed) by clustering analysis. A total of 38 differentially expressed proteins were screened in the SCV1 strain compared with the B1 strain. The ratio of protein expression level between SCV1 strain and B1 strain was standardized with ratio > 2 or ratio < 0.5 and *P-*value < 0.05 to define the differentially expressed proteins. All sequence hits of quantitative proteins were identified from the UniProtKB database.

To detect whether the adaptive characteristics were associated with the differential expression of OMPs, we performed quantitative proteomic analysis for the OMPs of the SCV1 and B1 strains. All quantitative protein sequence information was extracted from the UniProtKB database. The ratio of the protein expression level between the SCV1 and B1 strains was standardized with a ratio > 2 or ratio < 0.5 and a *P-*value < 0.05 used to define differentially expressed proteins. Thirty-eight differentially expressed proteins were identified in the SCV1 strain compared with the B1 strain (Figure 7, Table 1). Proteins that were upregulated in the SCV1 strain accounted for 36.84% (14/38) of differentially expressed proteins that were associated with membrane integrity, metabolic process, transferase activity, and transporter activity. An additional 24 downregulated proteins were involved in localization, transport, metabolism, cell motility and biological regulation.

**Table 1:** Differentially expressed proteins identified in the SCV1 strain by mass spectrometry analysis compared with the B1 strain.

**Locus_tag**	**Protein**	**Putative function**	**Fold change**
**UP-REGULATED PROTEINS**			
U876_04790		Trypsin-like serine protease	2.605
U876_01200	TorC	Cytochrome C	2.194
U876_00450	TetR	TetR family transcriptional regulator	2.598
U876_18555	CirA	TonB-dependent hemoglobin receptor family protein; outer membrane receptor proteins CirA	9.306
U876_02480		ABC-type transport auxiliary lipoprotein	2.016
U876_22720		Putative lipoproteins	3.255
U876_11065	Slp	Slp family outer membrane	2.010
U876_00470	LpsA	Beta-1,4-galactosyltransferase LPS and lipooligoaccharide biosynthesis protein	2.497
U876_13435	SurA	Surface antigen (outer membrane lipoprotein)	2.024
U876_14480	MalE	Sugar ABC transporter	3.046
U876_15120	LrgB	Murein hydrolase effector	2.565
U876_21525	LpoB	Outer membrane lipoprotein LpoB	4.748
U876_06385		Hypothetical protein	3.243
U876_18495	Lipo	Lipoprotein	2.291
**DOWN-REGULATED PROTEINS**			
U876_04930	RbfA	Ribosome-binding factor A	0.331
U876_07270	FlgE	Flagellar hook-filament junction protein	0.475
U876_07305	FlgL	Flagellar hook protein	0.241
U876_00240	EamA	EamA family transporter	0.447
U876_22450	GntP	Gluconate transporter	0.433
U876_21090	PiuB	Peptidase, iron-regulated membrane protein	0.345
U876_13395	CvpA	Bacteriocin production protein	0.473
U876_14050	SecD	Protein-export membrane protein	0.486
U876_14395	YbjE	Membrane protein	0.484
U876_05770	TppB	Peptide ABC transporter permease	0.150
U876_20365	Lgt	Prolipoprotein diacylglyceryl transferase	0.418
U876_19515	TorB	Cytochrome B	0.494
U876_21960	ChaA	Calcium: proton antiporter	0.272
U876_07035		UDP-phosphate alpha-N-acetylglucosaminyl 1-phosphate transferase	0.151
U876_10015		Inosine-5-monophosphate dehydrogenase	0.182
U876_04125		Hypothetical protein	0.398
U876_20330	MurJ	Multidrug transporter MviN/MurJ	0.295
U876_02390	NhaC	Antiporter	0.290
U876_RS16105		Hypothetical protein	0.258
U876_10370	PotE	Amino acid APC transporter	0.491
U876_08755	GltP	C_4_-dicarboxylate ABC transporter	0.485
U876_00825	NirC	Nitrite transporter	0.354
U876_02330	Pts IIBC	PTS system trehalose-specific transporter	0.322
U876_17165	Stpk	Serine/threonine protein kinase	0.401

### Validation of mass spectrometry results by qRT-PCR

**Figure 8:**
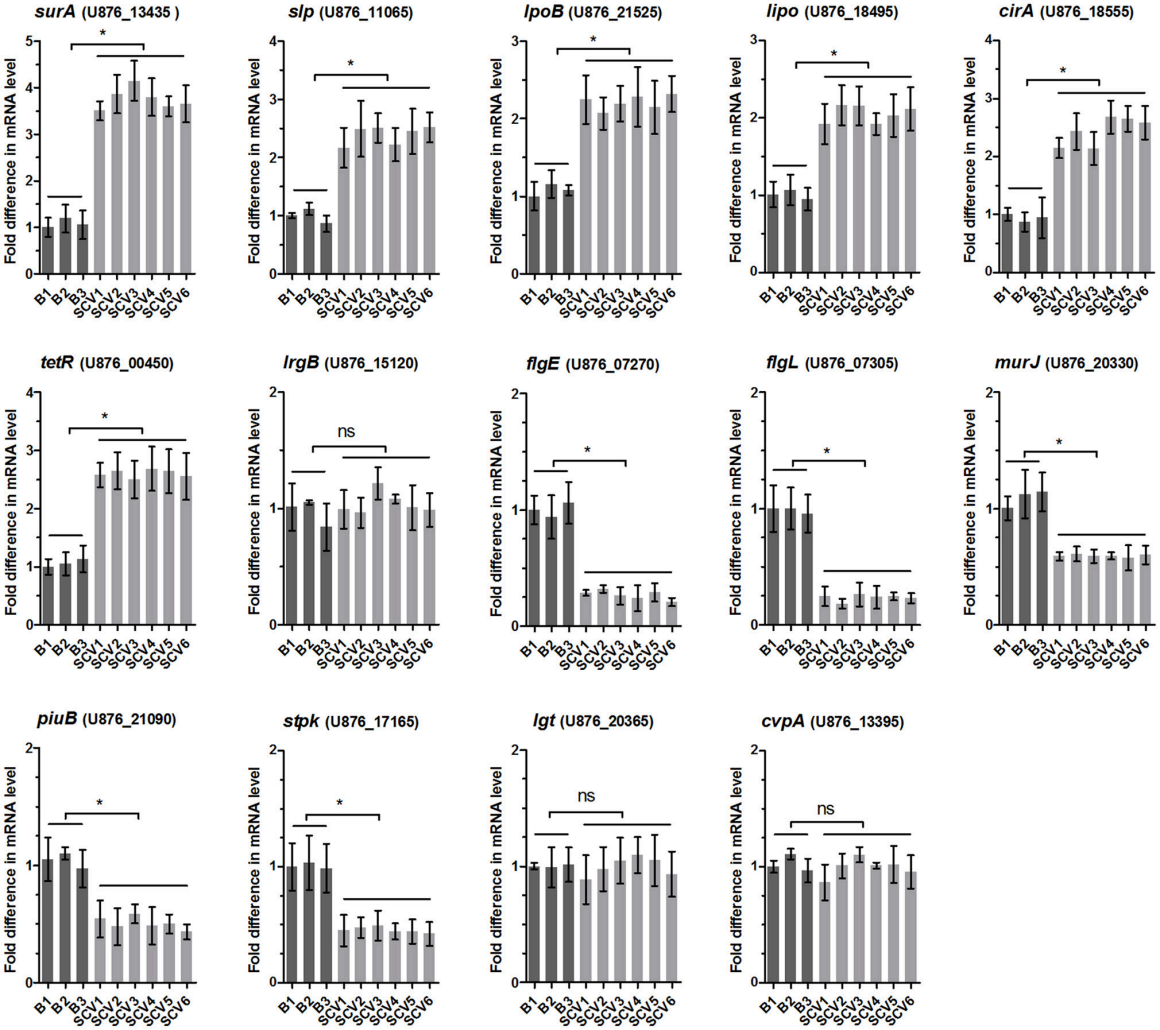
Relative mRNA expression levels of 14 genes coding the differentially expressed proteins in B strains (B1 thru B3) and SCV strains (SCV1 thru SCV6). For each sample, the acquired cycle threshold (CT) was normalized to the CT of the internal housekeeping gene *recA*, and the ΔCT was normalized to the ΔCT of the B1 strain. Relative fold differences in mRNA expression level were calculated using the 2^−ΔΔCT^ method, where ΔΔCT = (CT_geneofinterest_ – CT_*recA*gene_) all strain – (CT_geneofinterest_ – CT_*recA*gene_) B1. Data are presented as the mean ± SD of three independent experiments, with each experiment being comprised of four individual measurements. ^*^P < 0.05 indicates significant difference among different bacterial strains; ns indicates no significant difference.

To determine whether the differentially expressed proteins screened between the SCV1 and B1 strains applied to all of the isolated SCVs and B strains, the mRNA levels of 14 genes coding differently expressed proteins (SurA, Slp, LpoB, Lipo, CirA, TetR, LrgB, FlgE, FlgL, MurJ, PiuB, Stpk, Lgt, and CvpA) were measured by qRT-PCR in all of the B strains (B1 thru B3) and SCVs (SCV1 thru SCV6). As shown by Figure 8, the mRNA levels of *lrgB, lgt* and *cvpA* showed no alteration in SCVs as compared with the B strains, which might be due to the fact that post-transcriptional modifications play an important role in regulating the expression of the three genes. The other eleven genes were significantly upregulated or downregulated in all of the SCVs compared to the B strains, which was consistent with the mass spectrometry results.

### Roles of four differentially expressed proteins in fitness of *A. hydrophila*

**Figure 9:**
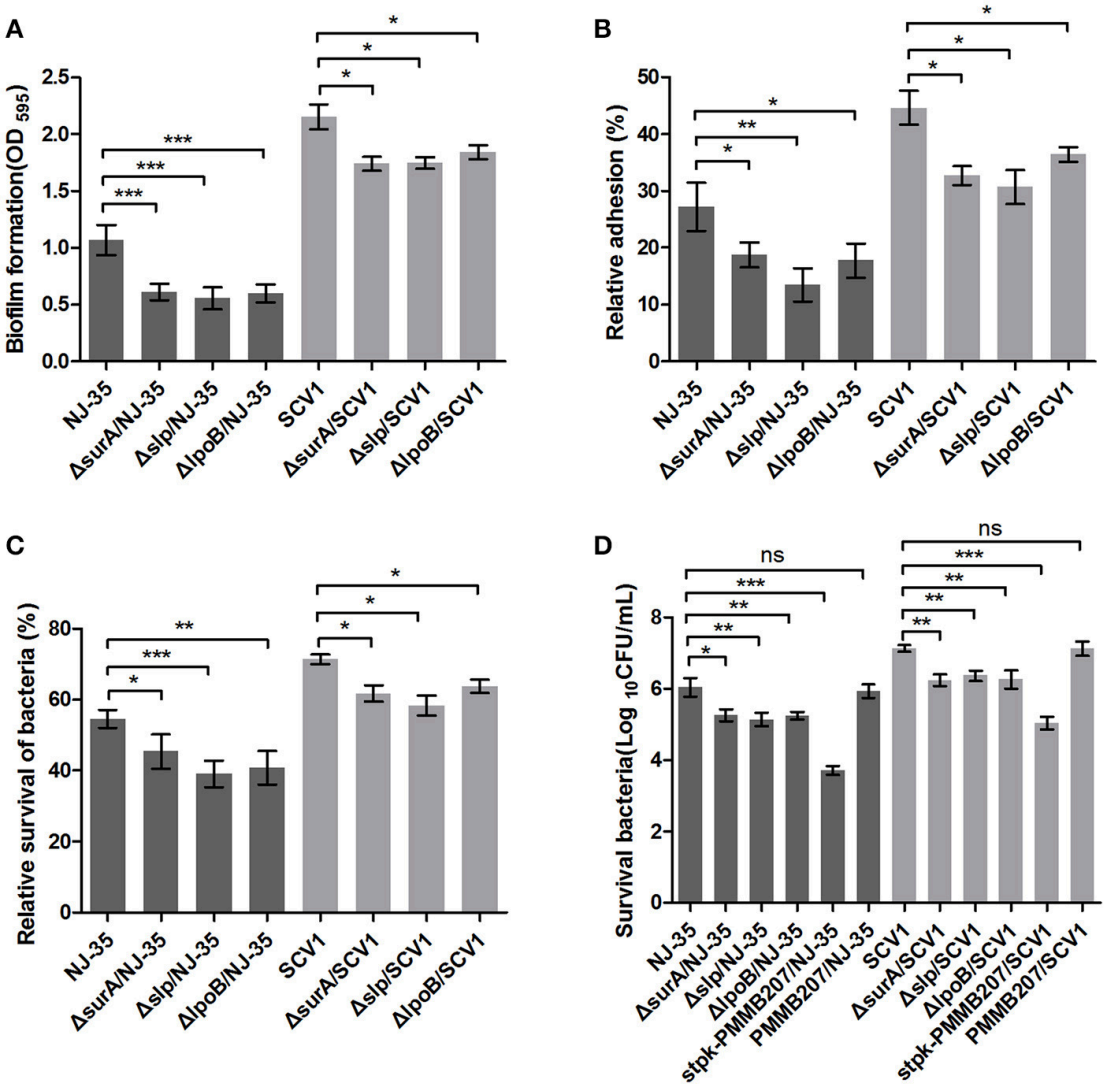
Characteristics of *surA, slp, lpoB* gene-deletion mutants and *stpk*-upregulated strains. **(A)** Biofilm formation of *A. hydrophila* NJ-35, SCV1 strain and the derived mutants. **(B)** Adhesion to HEp-2 cells of *A. hydrophila* NJ-35, SCV1 strain and the derived mutants. **(C)** Resistance to protistan predation of *A. hydrophila* NJ-35, SCV1 strain and the derived mutants. The relative survival of bacteria was expressed as the OD_450_ value of *A. hydrophila* co-cultured with *T. thermophila* divided by that of *A. hydrophila* grown alone at 12 h. **(D)** H_2_O_2_ resistance levels of *A. hydrophila* NJ-35, SCV1 strain, and their derived mutants and *stpk-*upregulated strains. Data are presented as the mean ± SD of four independent experiments, with each experiment being consisting of three replicates. ^*^P < 0.05, ^**^0.001 < *P* < 0.01 or ^***^P < 0.001 indicates significant difference compared with the parental strain; ns indicates no significant difference.

To determine whether the differentially expressed proteins SurA, Slp, LpoB, and Stpk play important roles in the environmental adaptability of *A. hydrophila* NJ-35, the gene deletion mutants Δ*surA*/NJ-35, Δ*slp*/NJ-35, Δ*lpoB*/NJ-35 and upregulated strain *stpk*-PMMB207/NJ-35 in *A. hydrophila* NJ-35, and Δ*surA*/SCV1, Δ*slp*/SCV1, Δ*lpoB*/SCV1 and *stpk*-PMMB207/SCV1 in SCV1 strain were constructed (Figure S1). The *surA, slp*, and *lpoB* gene-deletion mutants in both the NJ-35 and SCV1 strains displayed decreased biofilm formation (Figure 9A), decreased adhesion to HEp-2 cells (Figure 9B), decreased resistance to predation by *T. thermophila* (Figure 9C) and decreased resistance to oxidative stress (Figure 9D) when compared to their parental strain, respectively. In addition, the upregulated strains *stpk*-PMMB207/NJ-35 and *stpk*-PMMB207/SCV1 showed significantly increased sensitivity to H_2_O_2_ (Figure 9D). These results demonstrated that SurA, Slp, LpoB and Stpk play important roles in fitness of *A. hydrophila*.

### Effects of three lipoproteins on the virulence of SCVs and RAW264.7 macrophages

**Figure 10:**
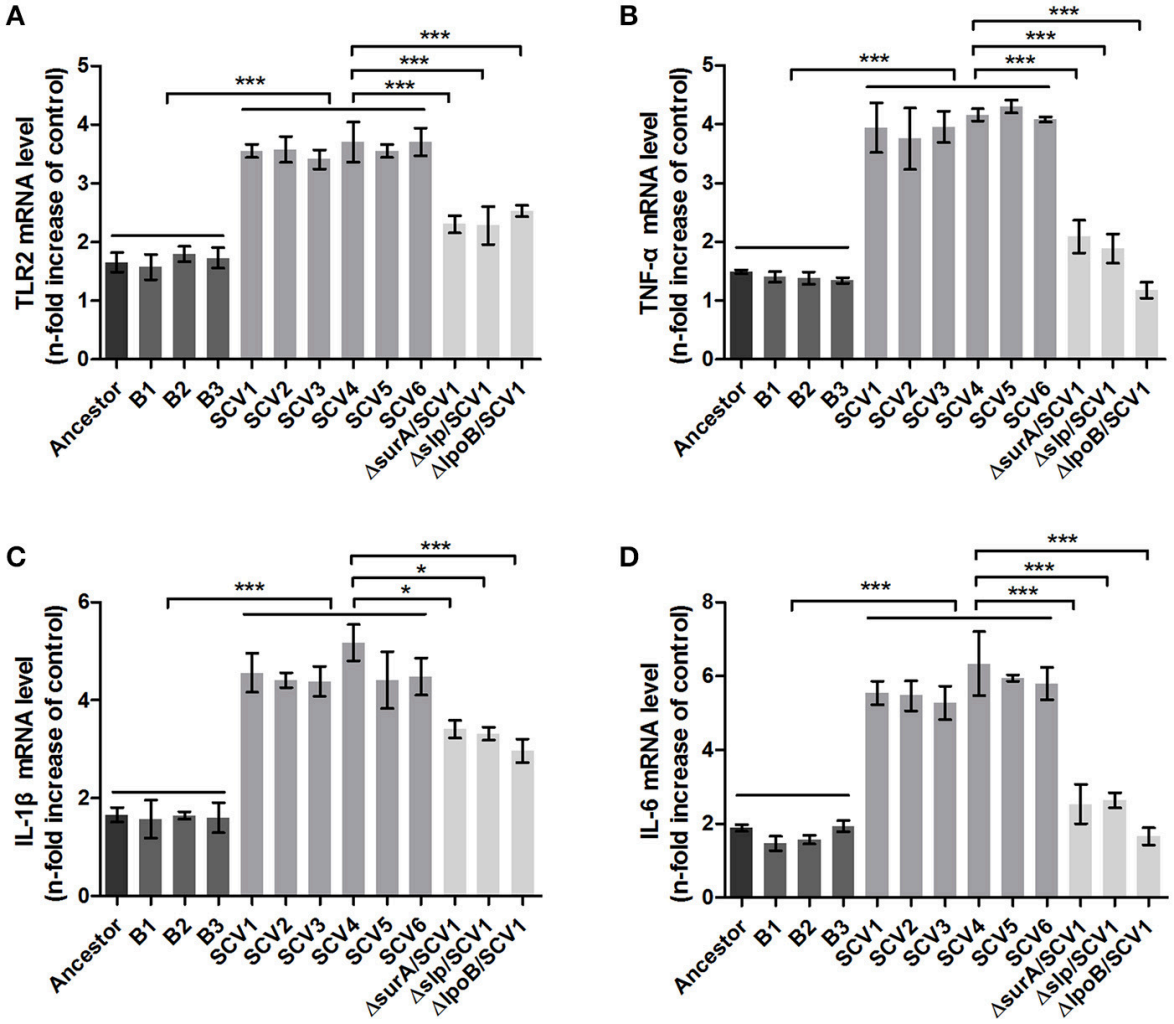
TLR2 and cytokines expression in RAW264.7 macrophages induced by *A. hydrophila* infection. RAW264.7 macrophages were infected with the ancestor strain, B strains, SCV strains, and Δ*surA*/SCV1, Δ*slp*/SCV1, and Δ*lpoB*/SCV1 mutants at a MOI of 1:1 for 1 h. After the addition of antibiotics for 3 h, the expression levels of TLR2 **(A)**, TNF-α **(B)**, IL-1β **(C)**, and IL-6 **(D)** were measured by qRT-PCR. Uninfected cells served as the control. The mRNA levels of TLR2, TNF-α, IL-1β, and IL-6 were normalized to those of β-actin and then were expressed as *n*-fold increases with respect to the control. Data are presented as the mean ± SD of three independent experiments, with each experiment being consisting of four replicates. ^*^P < 0.05, or ^***^P < 0.001 indicates significant difference among different bacterial strains.

Bacterial lipoproteins have been recognized to be involved in the host proinflammatory innate immune response mediated by Toll-like Receptor 2 (TLR2) (Kovacs-Simon et al., 2011; Sampson et al., 2013). To detect whether the attenuated virulence of SCVs in zebrafish were produced by lipoproteins through increasing proinflamatory response by TLR2, we evaluated the virulence of the three Δ*surA*/SCV1, Δ*slp*/SCV1, and Δ*lpoB*/SCV1 mutants in zebrafish and investigated the expression levels of TLR2, TNF-α, IL-1β, and IL-6 in response to infection with the mutants in RAW264.7 macrophages. As shown in Figure 6B, the LD_50_ values of the Δ*surA*/SCV1 (3.76 × 10^3^ CFU), Δ*slp*/SCV1 (3.03 × 10^3^ CFU) and Δ*lpoB*/SCV1 (4.50 × 10^3^ CFU) was lower than that of the SCV1 strain (9.29 × 10^3^ CFU), indicating an increase in the virulence of the mutants. In addition, the SCV strains caused an enhancement of mRNA levels of TLR2, TNF-α, IL-1β, and IL-6 in RAW264.7 cells compared to those of the ancestor and B strains (Figure 10). And infection of macrophages with the three Δ*surA*/SCV1, Δ*slp*/SCV1, and Δ*lpoB*/SCV1 mutants led to significantly decreased expression of TLR2, TNF-α, IL-1β, and IL-6 compared to those detected in the SCVs (Figure 10).

## Discussion

In natural habitats, bacteria are continuously challenged by various environmental factors, such as predation. *T. thermophila*, a bacterivorous predator, is very common in the aquatic environment, where *A. hydrophila* is often present. Thus, it is likely that the two organisms confront each other in the natural environment, and bacterial pathogenic mechanisms may be developed to resist predation or digestion by the predators, which will naturally affect the life cycle of the bacterium (Li et al., 2011; Pang et al., 2012). In this study, we showed that the predator-prey interaction between *T. thermophila* and *A. hydrophila* improved the environmental adaption of *A. hydrophila*. After semi-continuous subculture with *Tetrahymena*, a majority of *A. hydrophila* isolates were SCVs. Colony morphology variation has been considered to be an evolutionary process that is used to overcome stressful conditions (Sousa et al., 2011). Besides, the morphological adaptations such as forming cell aggregates (Corno et al., 2013; Baumgartner et al., 2016) have also been reported to be the possible strategies to protect bacteria from protistan grazing. Consistent with this, our study showed that the SCVs displayed cell aggregates, which was different from the B strains. To evaluate the correlation between morphology variation and environmental adaptation of *A. hydrophila*, we investigated several features associated with bacterial survival strategies in the SCVs.

Bacterial communities, such as biofilms, are demonstrated to play an important role in their survival and persistence in harsh environments by providing a number of pathogens with an adaptive advantage (Parsek and Singh, 2003; Johnson, 2008). In this study, the co-culture of *A. hydrophila* and *T. thermophila* notably increased biofilm formation in the resulting *A. hydrophila* isolates. This finding is consistent with a previous report in which the grazing activity of *Cafeteria roenbergensis* on planktonic *Vibrio cholerae* was observed to stimulate the formation of grazing-resistant biofilms (Matz et al., 2005). Additionally, the SCVs in our study displayed increased cell adhesion, which has been showed to be associated with biofilm formation (Wang et al., 2014). The OMPs are one type of the adhesins in *Aeromonas* (Tomás, 2012) and have been reported to play important roles in bacterial biofilm formation (Chen et al., 2017; Llama-Palacios et al., 2017). To investigate the possible mechanisms for the enhanced biofilm formation and adhesion in the SCVs, we performed a comparative proteomics analysis of OMPs between the SCV1 and B1 strains. Three outer membrane lipoproteins, including SurA, Slp and LpoB, were found to be upregulated in the SCV1. Then the mRNA levels of the three lipoprotein genes were demonstrated to be elevated in all the six SCVs. Further, the inactivation of the three genes in both the ancestor and SCV1 lead to a decrease in biofilm formation and adhesion, indicating that the three lipoproteins contribute to biofilm formation and adhesion of the SCVs.

Bacterial motility provides a survival advantage under various environments, allowing bacteria to compete successfully for nutrients (Matz and Jürgens, 2005). However in this study, the SCVs showed decreased swimming and swarming abilities. We speculate that the decreased motility may be an adaptive trait of *A. hydrophila* to escape predation. Gonzalez et al. (1993) reported that motile bacteria were more susceptible to be preyed upon than non-motile strains in aquatic laboratory microcosms. Motile bacterial strains would experience an increased rate of encounters (contact) with protists (Matz et al., 2002), which appears to be the cause for the more rapid clearance of motile bacteria than that of non-motile cells (Gonzalez et al., 1993). The decreased motility of the SCVs might be due to limited polar flagella synthesis, which might be mediated by the downregulation of the flagellar hook proteins FlgE and FlgL that are responsible for bacterial motility (Wozniak et al., 2010; Moriya et al., 2013). Of note, our previous study reported that *A. hydrophila* NJ-35 possesses only a single polar flagellum, whereas the lateral flagella is absent (Pang et al., 2015). It is known that a single flagellum is sufficient for swimming motility, but most bacteria that swarm require lateral flagella to contact with surfaces (Kearns, 2010). However, in our present and previous studies (Pang et al., 2017), *A. hydrophila* NJ-35 was demonstrated to have the swarming ability. Similar phenomenon has been found in *Pseudomonas aeruginosa*, which could synthesize an alternative motor (Toutain et al., 2005) or produce two polar flagella (Köhler et al., 2000) during swarming to propel movement on surfaces.

In addition to protistan predators, lytic bacteriophages are another major biotic cause of bacterial mortality in aquatic environments (Zhang et al., 2014b). Bacteria evolve resistance to phage infection for their survival. A previous study showed that *Serratia marcescens* and *Pseudomonas* isolates having been exposed to protist predators exhibited decreased susceptibility to infections by lytic phages (Örmälä-Odegrip et al., 2015). After being semi-continuously cocultured with ciliates, *A. hydrophila* had displayed a decreased susceptibility to lytic phages. Further adsorption kinetics assays revealed that adsorption inhibition might be a potential cause for the phage resistance, but the mechanism by which this inhibition occurs is unclear. Labrie et al. (2010) suggested that the mechanisms of phage adsorption inhibition can be divided into three categories: the blocking of phage receptors, the production of extracellular matrix and the production of competitive inhibitors. In this study, electron microscopic observation indicated that the SCVs were enveloped by secretions (Figure S2), so we speculate that extracellular matrix may have covered the phage receptor and limited phage adsorption. Additionally, Labrie et al. (2010) demonstrated that bacteria could use lipoproteins to adaptively alter the structure or three-dimensional conformation of phage receptors to inhibit phage adsorption. The outer-membrane lipoprotein TraT of *E. coli* has been reported to block or modify the conformation of the outer-membrane protein A (OmpA), which serves as a receptor for many phages (Riede and Eschbach, 1986). In this study, six putative lipoproteins were observed to be upregulated in the SCVs. Whether the lipoproteins are involved in the inhibition of phages adsorption requires further study to be verified.

Bacterial responses to environmental stresses are crucial for their growth, survival, and adaptation. Our study indicated that the isolated SCVs harbored resistance to H_2_O_2_ and increased survival against *E. coli*. The environmental adaptation might be associated with the altered expression of a broad set of proteins, as indicated by our proteomics data. For example, serine/threonine protein kinases (STPKs) have been shown to be involved in stress responses in prokaryotes (Av-Gay and Everett, 2000). The inactivation of *pknE, pknI, pknK*, or *pknL*, which encode STPKs in *Mycobacterium tuberculosis*, resulted in an increased survival in response to an acidic pH, oxidative pressure, and the presence of lysozyme and antibiotics (Gopalaswamy et al., 2009; Malhotra et al., 2010; Kumar et al., 2013; Refaya et al., 2016). Consistent with this, a Stpk protein was detected to be down-regulated in the SCVs. And the *stpk*-overexpression strains displayed decreased survival after treatment of H_2_O_2_. In addition, the *surA, slp*, and *lpoB* gene-deletion mutants also showed decreased resistance to H_2_O_2_. This led to us to conclude that the upregulated SurA, Slp, and LpoB, and the downregulated Stpk observed in this study might be involved in the resistance of the SCVs to adverse environmental conditions.

Biofilm formation, adhesion, anti-oxidative stress and inter-specific competition are known to contribute to bacterial virulence (Parsek and Singh, 2003; Zheng et al., 2011; Kishikawa et al., 2013; Romsang et al., 2013; Singh et al., 2015). However, the virulence of SCVs in this study was significantly attenuated in zebrafish, which appeared to be contrary to published studies. Although a similar phenomenon has been reported in which ciliates were observed to select for attenuated virulence of *S. marcescens* but resulted in enhanced persistence in the environment (Friman et al., 2009; Mikonranta et al., 2012; Zhang et al., 2014a), the molecular mechanisms are still unclear. We speculated that under predation pressure, *A. hydrophila* might evolve strategies to subvert host defense response aimed at combating pathogens. To be noted, lipoproteins such as SurA, Slp and LpoB were upregulated in the SCVs. Bacterial lipoproteins have been demonstrated to enhance host innate immune response through Toll-like receptor 2 (TLR2) activation, which can stimulate the production of proinflammatory cytokines such TNF-α, IL-1β, and IL-6 (Seya and Matsumoto, 2002; Kovacs-Simon et al., 2011; Sampson et al., 2013). To determine whether the attenuated virulence of the SCVs in zebrafish were caused by lipoproteins due to immune response mediated by TLR2, we evaluated the virulence of the three mutants Δ*surA*/SCV1, Δ*slp*/SCV1, and Δ*lpoB*/SCV1 in zebrafish and investigated the expression levels of TLR2, TNF-α, IL-1β, and IL-6 in RAW264.7 macrophages in response to infection with the mutants. Interestingly, the results indicated that the virulence of the three mutants were increased compared to the SCVs (Figure 6B). Furthermore, the SCVs caused an obvious enhancement of mRNA levels of TLR2, TNF-α, IL-1β, and IL-6 in RAW264.7 cells compared to those of the ancestor and B strains (Figure 10). And infection of macrophages with the three Δ*surA*/SCV1, Δ*slp*/SCV1, and Δ*lpoB*/SCV1 mutants led to significantly decreased expression of TLR2, TNF-α, IL-1β, and IL-6 compared with those detected in the SCVs (Figure 10). The results indicated that the attenuated virulence of the SCVs in zebrafish could be due, at least in part, to the high levels of cytokines produced in response to infection through TLR2 recognizing up-regulated lipoproteins.

To be noted, our study mainly focused on the proteins that might be associated with the increased fitness of the SCVs. However, genome reduction has been considered to be a consequence of predation selection to increase bacterial survival and fitness (Koskiniemi et al., 2012; Baumgartner et al., 2017). In this regard, whether the reduction of genome size in the SCVs evolved with predator need our further investigation.

In conclusion, our results demonstrated that the predation of *T. thermophila* accelerates the adaptive evolution of *A. hydrophila* NJ-35, resulting in the alteration of diverse traits. The observed alterations in bacterial characteristics contribute to our understanding of defensive and adaptive mechanisms *A. hydrophila* exhibits in the environment. The differentially expressed proteins identified in this study suggest that many proteins might be associated with the defense of *A. hydrophila* with respect to predation. Especially, three lipoproteins SurA, Slp and LpoB and a serine/threonine protein kinase (Stpk) play important roles in environmental adaptation and/or attenuated virulence of *T. thermophila*-exposed strains. This study provides an important contribution to the understanding of the defensive traits of *A. hydrophila* against protistan predators.

## Author contributions

YL and JL conceived the study, interpreted the data and drafted the paper; JL, YD, and NW performed most of the experiments described in the manuscript; SL, YY, YW, and FA helped with the experiments; CL provided valuable suggestions of the manuscript.

### Conflict of interest statement

The authors declare that the research was conducted in the absence of any commercial or financial relationships that could be construed as a potential conflict of interest.
