# Supplementary material for: Tetrahymena thermophila Predation Enhances Environmental Adaptation of the Carp Pathogenic Strain Aeromonas hydrophila NJ-35
Source: Front Cell Infect Microbiol. 2018 Mar 14;8:76. doi: 10.3389/fcimb.2018.00076 (PMC5861188; doi:10.3389/fcimb.2018.00076)
Supplement: Supplementary file 1 [file Table1.DOC]

**Table S1** Primers of 14 differently expressed genes used for real time PCR

| **Target gene** | **Locus_tag** | **Primers** | **Sequences (5’-3’)** |
| --- | --- | --- | --- |
| **Up-regulated proteins** | | | |
| **Slp** | U876_11065 | *slp*-F | GTGGTCTATCTGCCGCTCAA |
|  |  | *slp*-R | TGCTCGCCTATCTGGCTGTC |
| **Lipo** | **U876_18495** | ***lipo***-F | GCAGCAAGGATCTGAGCGAT |
|  |  | ***lipo***-R | AGGGCGTTGAGCATCTTGG |
| **SurA** | U876_13435 | ***surA***-F | CACCGACCGCAGTAGCAAT |
|  |  | ***surA***-R | CAACATCACCCAGCGTCAAAT |
| **LpoB** | U876_21525 | ***lpoB***-F | GAGCAAAAGCGGTTTTCGC |
|  |  | ***lpoB***-R | CCAGATCCCCGTAGAGCATGT |
| **CirA** | U876_18555 | *cirA*-F | CAGTGCATCGGAGCCGTAA |
|  |  | *cirA*-R | CCAGACCTTCGTCATTCGTG |
| **TetR** | U876_00450 | ***tetR***-F | ATTGGCGTAGAAGAAGCGGA |
|  |  | ***tetR***-R | CGGGCAACCTCTATTACCACT |
| **LrgB** | U876_15120 | ***lrgB***-F | TCATCGTCGGCATCATAGGC |
|  |  | ***lrgB***-R | CTCGCTGGTGAAGAACTCGG |
| **Down-regulated proteins** | | | |
| **FlgE** | U876_07270 | ***flgE***-F | ATCAACACGGATGGGACGC |
|  |  | ***flgE***-R | GCTCGGTCATTTTTGGCTCA |
| **FlgL** | U876_07305 | ***flgL***-F | ATCAGGGCAACTTCGACAGC |
|  |  | ***flgL***-R | CAACAAGGCACCACCTCCAT |
| **PiuB** | U876_21090 | ***piuB***-F | TGGCACTTCTATGCGGGTCT |
|  |  | ***piuB***-R | GCTCGGGGTACATCAGGTTG |
| **MurJ** | U876_20330 | ***murJ***-F | GCAGTCGGTCCGAGTAATAGAG |
|  |  | ***murJ***-R | GGTGCAGTTCCTGTTCCAGTT |
| **Lgt** | U876_20365 | ***lgt***-F | GGTGAAGAAGTGGCGGTTG |
|  |  | ***lgt***-R | GCTGTTTTACGGCTTCCTCG |
| **CpvA** | U876_13395 | *cvpA*-F | ACAGCCCCGTCTTGTCTACC |
|  |  | *cvpA* -R | CTCGCCATCTACTTCACCTCC |
| **Stpk** | U876_17165 | ***stpk***-F | GTTCTACCTGATCCCGCACTG |
|  |  | ***stpk***-R | ACCTTCACCGTAACGACCCTT |
| **Reference gene** | | | |
| recA | U876_02875 | *recA*-F | CGACCCCATCTATGCCGC |
|  |  | *recA*-R | CCATCTCACCTTCGATTTCCG |
