# Supplementary material for: Tetrahymena thermophila Predation Enhances Environmental Adaptation of the Carp Pathogenic Strain Aeromonas hydrophila NJ-35
Source: Front Cell Infect Microbiol. 2018 Mar 14;8:76. doi: 10.3389/fcimb.2018.00076 (PMC5861188; doi:10.3389/fcimb.2018.00076)
Supplement: Supplementary file 2 [file Table2.DOC]

**Table S2** Primers used for construction and confirmation of *surA, slp, lpoB* gene-deletion mutants of *A .hydrophila* NJ-35

| **Target gene** | **Length**  **(bp)** | **Primer** | | **Sequence(5’-3’)** | |  |
| --- | --- | --- | --- | --- | --- | --- |
| Construction of *surA* deletion mutant | | | | | |  |
| *surA* -upstream | 759 | *surA*-P1 | | CAGGTCGACTCTAGAGGATCCCATCGGCATCTCGGTGTC (*Bam*HI) | |  |
|  |  | *surA* -P2 | | AGGTTCACAGAGCAGGCGCTGGCTCA | |  |
| *surA* -downstream | 682 | *surA* -P3 | | AGCGCCTGCTCTGTGAACCTCGTTACCTTATG | |  |
|  |  | *surA* -P4 | | GAGCTCGGTACCCGGGGATCCTTGTCGGCTCAGTTGTGC (*Bam*HI) | |  |
| *surA* (partial) | 361 | *surA* -a | | CACGCCATCGTCGAAATA | |  |
|  |  | *surA* -b | | GCCCGTGAATCAGGTGG | |  |
| Construction of *slp* deletion mutant | | | | | |  |
| *slp* -upstream | 661 | *slp*-P1 | | CAGGTCGACTCTAGAGGATCCAGGCTGTGTTGGACGAC (*Bam*HI) | |  |
|  |  | *slp*-P2 | | ACTCCCGGTCGTCACGCTCCTCTGGC | |  |
| *slp* -downstream | 784 | *slp*-P3 | | GGAGCGTGACGACCGGGAGTGAAGACC | |  |
|  |  | *slp*-P4 | | GAGCTCGGTACCCGGGGATCCTCTATCTGGGCAAACGC (*Bam*HI) | |  |
| *slp* (partial) | 478 | *slp* -a | | GTTGAAGCGTGCAATGGT | |  |
|  |  | *slp* -b | | ATAGAAGGGGTCGTAGAAGTAG | |  |
| Construction of *lpoB* deletion mutant | | | | | |  |
| *lpoB* -upstream | 550 | *lpoB*-P1 | | CAGGTCGACTCTAGAGGATCCGCATTGAGTCTGAGCGGTT (*Bam*HI) | |  |
|  |  | *lpoB* -P2 | | GAGAGAAAAGGCAGCCAGCGATCCAT | |  |
| *lpoB* -downstream | 578 | *lpoB* -P3 | | CGCTGGCTGCCTTTTCTCTCCGCCAG | |  |
|  |  | *lpoB* -P4 | | GAGCTCGGTACCCGGGGATCCCTTCCAAATCCGCAGT (*Bam*HI) | |  |
| *lpoB* (partial) | 514 | *lpoB* -a | | TCAGGGTCATCGACAGCC | |  |
|  |  | *lpoB* -b | | TCCTTGTTGATGGTGGGTG | |  |
| Construction of *stpk* upregulation mutant | | | | | |  |
| *Stpk* gene plus  its promoter and terminator regions | 1937 | *stpk-F* | | GAGCTCGGTACCCGGGGATCCTTCACAAAATTGGGCTCG (*Bam*HI) | |  |
|  |  | *stpk-R* | | CAGGTCGACTCTAGAGGATCCGGTAATGAACGAAGCCAAAC (*Bam*HI) | |  |
| region containing two flanking of *Bam*HI in plasmid PMMB207 | 733 | | PMMB207-F | | CGTTACTGGTTTCACATTCAC | |
|  |  |  | PMMB207-R | | GTCCTACTCAGGAGAGCGTT | |
