# Supplementary material for: Tetrahymena thermophila Predation Enhances Environmental Adaptation of the Carp Pathogenic Strain Aeromonas hydrophila NJ-35
Source: Front Cell Infect Microbiol. 2018 Mar 14;8:76. doi: 10.3389/fcimb.2018.00076 (PMC5861188; doi:10.3389/fcimb.2018.00076)
Supplement: Supplementary file 3 [file Table3.DOC]

**Table S3** Primers used for qRT-PCR after macrophage infections and stimulations

| **Primer** | **Sequence(5’-3’)** | Function |
| --- | --- | --- |
| TLR2-F | TCTACGGGCAGTGGTGAAAAC | Fragment for mouse TLR-2 gene |
| TLR2-R | ATCAGTCCCAAAGTCTAAAGTCG |
| TNF-α-F | AAGCCTGTAGCCCACGTCGTA | Fragment for mouse TNF-α gene |
| TNF-α-R | GGCACCACTAGTTGGTTGTCTTTG |
| IL-1β-F | TCCAGGATGAGGACATGAGCAC | Fragment for mouse IL-1β gene |
| IL-1β-R | GAACGTCACACACCAGCAGGTTA |
| IL-6-F | CCACTTCACAAGTCGGAGGCTTA | Fragment for mouse IL-6 gene |
| IL-6-R | GCAAGTGCATCATCGTTGTTCATAC |
| β-actin-F | TGACAGGATGCAGAAGGAGA | Fragment for mouse β-actin gene |
| β-actin-R | GCTGGAAGGTGGACAGTGAG |
