# Supplementary figures and images for: Tetrahymena thermophila Predation Enhances Environmental Adaptation of the Carp Pathogenic Strain Aeromonas hydrophila NJ-35
Source: Front Cell Infect Microbiol. 2018 Mar 14;8:76. doi: 10.3389/fcimb.2018.00076 (PMC5861188; doi:10.3389/fcimb.2018.00076)

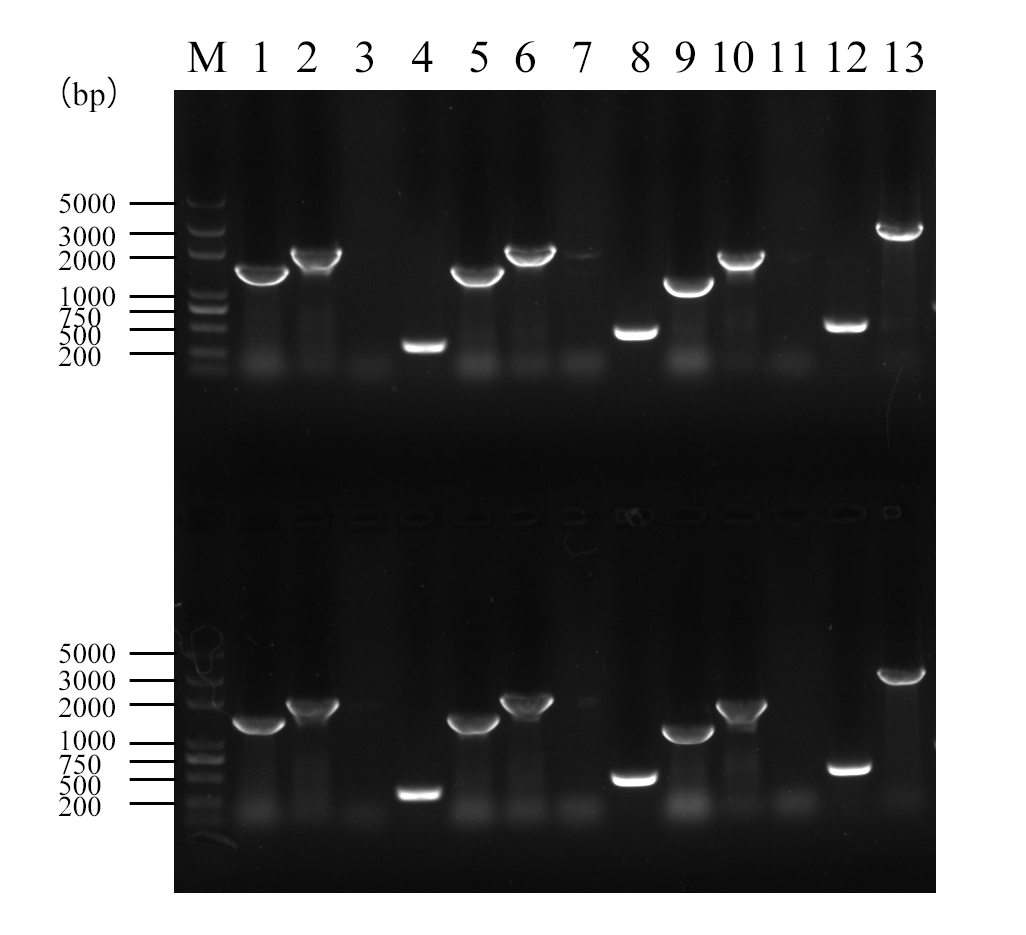

Supplement: Supplementary file 4 [file Presentation1.ZIP › Supplementary Figures/Supplementary Figure S1.tif]

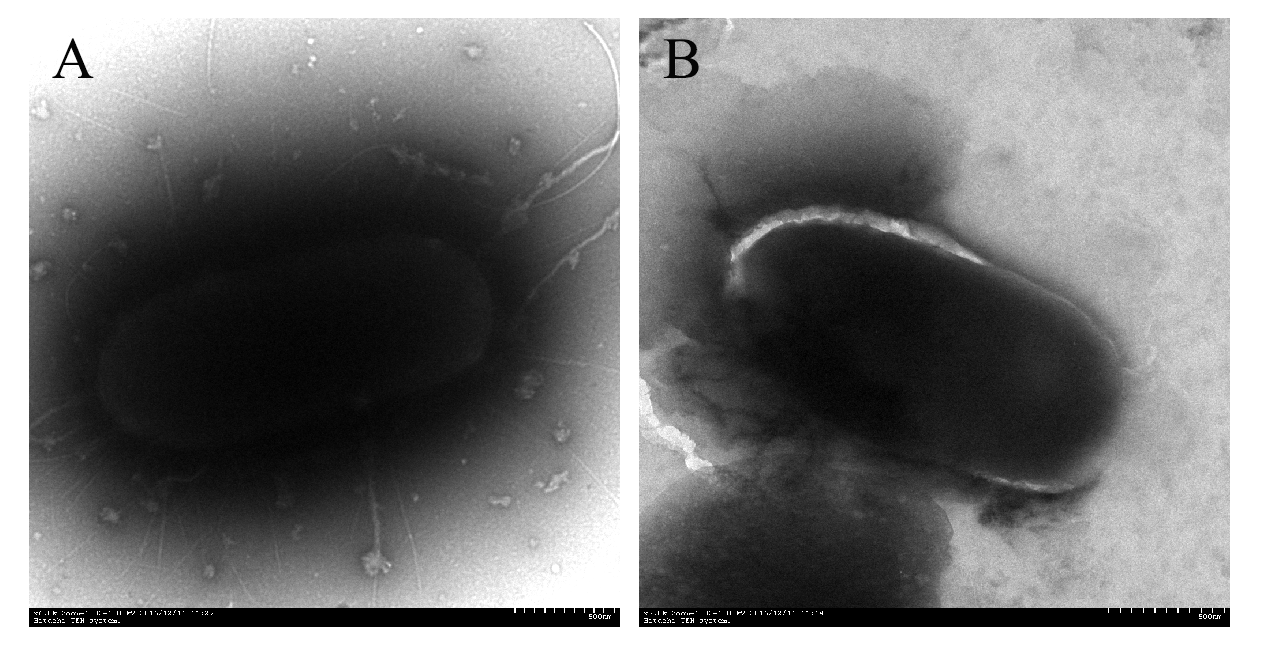

Supplement: Supplementary file 4 [file Presentation1.ZIP › Supplementary Figures/Supplementary Figure S2.tif]
