# Supplementary material for: Tetrahymena thermophila Predation Enhances Environmental Adaptation of the Carp Pathogenic Strain Aeromonas hydrophila NJ-35
Source: Front Cell Infect Microbiol. 2018 Mar 14;8:76. doi: 10.3389/fcimb.2018.00076 (PMC5861188; doi:10.3389/fcimb.2018.00076)
Supplement: Supplementary file 4 [file Presentation1.ZIP › Supplementary Figures/Supplementary_Material _Figures.doc]

Supplementary Material

***Tetrahymena thermophila* predation enhances environmental adaptation of the carp pathogenic strain *Aeromonas hydrophila* NJ-35**

Jin Liu, Yuhao Dong, Nannan Wang, Shougang Li, Yuanyuan Yang, Yao Wang, Furqan Awan, Chengping Lu and Yongjie Liu*

College of Veterinary Medicine, Nanjing Agricultural University, Nanjing, China

***Correspondence:** Yongjie Liu

E-mail: liuyongjie@njau.edu.cn;

**Supplementary Figures**

**Figure S1.** Identification of Δ*surA*, Δ*slp* and Δ*lpoB* gene-deletion mutants and stpk-upregulation strains by PCR amplification. M, DL5000 DNA marker; Lane 1 and 2 represent the *surA* gene plus its upstream and downstream was detected using primer *surA*-P1/P4 in the NJ-35 (and BT1) (1912 bp) and Δ*surA* (1441bp ) respectively; Lane 3 and 4 represent the *surA* gene was detected using primer *surA*-a/b in the NJ-35 (and BT1) (361bp) and Δ*surA* (none) respectively. Lane 5 and 6 represent the *slp* gene plus its upstream and downstream was detected using primer *slp*-P1/P4 in the NJ-35 (and BT1) (1961bp ) and Δ*slp* (1445 bp) respectively; Lane 7 and 8 represent the *slp* gene was detected using primer *slp*-a/b in the NJ-35 (and BT1) (478bp) and Δ*slp* (none) respectively. Lane 9 and 10 represent the *lpoB* gene plus its upstream and downstream was detected using primer *lpoB* -P1/P4 in the NJ-35 (and BT1) (1710 bp) and Δ*lpoB* (1128 bp) respectively; Lane 11 and 12 represent the *lpoB* gene was detected using primer *lpoB* -a/b in the NJ-35 (and BT1) (514bp) and Δ*lpoB* (none) respectively; Lane 11 and 12 represent the *stpk-*PMMB207 was detected using primer PMMB207 -F/R in the NJ-35 (and BT1) (733bp) and *stpk-*PMMB207/NJ-35 (*stpk-*PMMB207/BT1) (2670bp) respectively.

**Figure S2.** Observation through Transmission electron microscopy (TEM). (A) B (non-*Tetrahymena-*exposed) strain. (B) BT (*Tetrahymena*exposed) strain was enveloped by secretions.
